# The Crohn’s Disease Risk Factor IRGM Limits NLRP3 Inflammasome Activation by Impeding Its Assembly and by Mediating Its Selective Autophagy

**DOI:** 10.1016/j.molcel.2018.11.018

**Published:** 2019-02-07

**Authors:** Subhash Mehto, Kautilya Kumar Jena, Parej Nath, Swati Chauhan, Srinivasa Prasad Kolapalli, Saroj Kumar Das, Pradyumna Kumar Sahoo, Ashish Jain, Gregory A. Taylor, Santosh Chauhan

**Affiliations:** 1 Cell Biology and Infectious Diseases Unit, Institute of Life Sciences, Bhubaneswar 751023, India; 2 School of Biotechnology, KIIT University, Bhubaneswar 751024, India; 3 Department of Molecular Cell Biology, Institute for Cancer Research, Oslo University Hospital, Oslo, Norway; 4 Centre for Cancer Cell Reprogramming, Institute of Clinical Medicine, Faculty of Medicine, University of Oslo, Oslo, Norway; 5 Geriatric Research, Education, and Clinical Center, VA Medical Center, Durham, NC 27705, USA

**Keywords:** IRGM, Irgm1, NLRP3, ASC, autophagy, p62, inflammasome, Crohn’s disease, inflammatory disorders, autoimmunity, inflammatory bowel diseases

## Abstract

Several large-scale genome-wide association studies genetically linked IRGM to Crohn’s disease and other inflammatory disorders in which the IRGM appears to have a protective function. However, the mechanism by which IRGM accomplishes this anti-inflammatory role remains unclear. Here, we reveal that IRGM/Irgm1 is a negative regulator of the NLRP3 inflammasome activation. We show that IRGM expression, which is increased by PAMPs, DAMPs, and microbes, can suppress the pro-inflammatory responses provoked by the same stimuli. IRGM/Irgm1 negatively regulates IL-1β maturation by suppressing the activation of the NLRP3 inflammasome. Mechanistically, we show that IRGM interacts with NLRP3 and ASC and hinders inflammasome assembly by blocking their oligomerization. Further, IRGM mediates selective autophagic degradation of NLRP3 and ASC. By suppressing inflammasome activation, IRGM/Irgm1 protects from pyroptosis and gut inflammation in a Crohn’s disease experimental mouse model. This study for the first time identifies the mechanism by which IRGM is protective against inflammatory disorders.

## Highlights

• IRGM negatively regulates NLRP3/ASC inflammasome activation
• IRGM obstructs NLRP3/ASC inflammasome assembly
• IRGM mediates selective autophagic degradation of NLRP3/ASC inflammasome
• Irgm1 by suppressing Nlrp3 inflammasome prevents gut inflammation in an IBD mouse model

## Graphical Abstract

**Figure:**
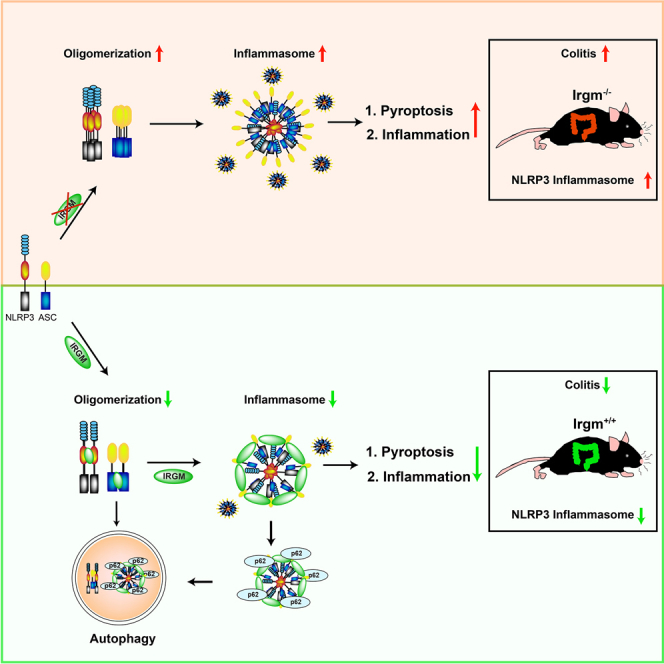

## Teaser

The IRGM is an established genetic risk factor for Crohn’s diseases and several other inflammatory disorders. Mehto et al. show that IRGM inhibits NLRP3 inflammasome activation to keep the inflammation under check. This study provides a basis for the protective function of IRGM in inflammatory diseases.

## Introduction

Acute inflammation is an essential innate immune response to self-protect from harmful stimuli, including irritants and pathogens. However, a chronic inflammatory response is deleterious and leads to several inflammatory diseases (Okin and Medzhitov, 2012). Autophagy is a fundamental cell-survival process that plays a broad homeostatic role in cleaning cells by removing toxic wastes including determinants of surplus inflammation (Deretic et al., 2013, Ma et al., 2013). Recently, several studies have demonstrated the importance of autophagy in protecting against chronic inflammatory diseases (Deretic and Klionsky, 2018).

One of the most critical components of the inflammatory response are inflammasomes, which are molecular complexes that are activated by diverse danger signals of pathogenic and non-pathogenic origin, resulting in the production of the pro-inflammatory cytokines such as interleukin-1β (IL-1β) and IL-18 (Jo et al., 2016, Schroder et al., 2018). A well-studied inflammasome is composed of the NLRP3 (NACHT, LRR, and PYD domains containing protein 3) protein that complexes with ASC (PYCARD) (apoptosis-associated speck-like protein containing a CARD) to form a caspase-1-activating complex that then cleaves and activates IL-1β (Guo et al., 2015). Dysregulation of NLRP3 inflammasome has been linked with several chronic inflammatory, infectious, and autoimmune diseases (Lamkanfi and Dixit, 2012, Strowig et al., 2012). Autophagy has emerged as one of the essential processes in controlling excess inflammasome activation. By mediating the degradation of obsolete mitochondria and by degrading inflammasome components including NLRP3, autophagy keeps unnecessary and aberrant activation of inflammasomes under check (Shi et al., 2012, Yan et al., 2015, Zhong et al., 2016).

Immunity-related GTPase M (IRGM) belongs to a family of interferon-inducible GTPases (IRGs), one of the strongest cell-autonomous resistance systems to intracellular pathogens (Hunn et al., 2011). After a landmark study showing the importance of human IRGM in anti-mycobacterial autophagy (Singh et al., 2006), several genome-wide association studies identified SNPs in the *IRGM* gene and a deletion polymorphism in *IRGM* promoter region as being strongly associated with Crohn’s disease (CD) and tuberculosis (Brest et al., 2011, Che et al., 2010, McCarroll et al., 2008, Parkes et al., 2007, Wellcome Trust Case Control, 2007, Craddock et al., 2010). Later, IRGM was genetically and functionally linked with several other chronic inflammatory and autoimmune diseases (Baskaran et al., 2014, Burada et al., 2012, Glas et al., 2013, Yang et al., 2014). Given the linkage of IRGM with so many inflammatory and autoimmune disorders, it is surprising that IRGM’s mechanism of action in regulating inflammation remains unclear.

In this study, our work reveals that human IRGM and its mice ortholog Irgm1 control inflammation by suppressing the activation of NLRP3 inflammasomes. Mechanistically, we found that IRGM physically complexes with NLRP3 inflammasome components and obstructs inflammasome assembly. IRGM interacts with SQSTM1/p62 (henceforth, p62) and mediates p62-dependent selective autophagy of NLRP3 and ASC. Thus, by restricting inflammasome activity, IRGM protects from pyroptosis. Further, we found that mouse Irgm1 suppresses the colon inflammation by inhibiting NLRP3 inflammasome in a DSS-induced colitis mouse model. Taken together, this work identifies a direct role of IRGM in suppressing the inflammation and provides a basis for its protective role in inflammatory diseases including Crohn’s.

## Results

### Human IRGM Suppresses Pro-inflammatory Cytokine Response

**Figure 1:**
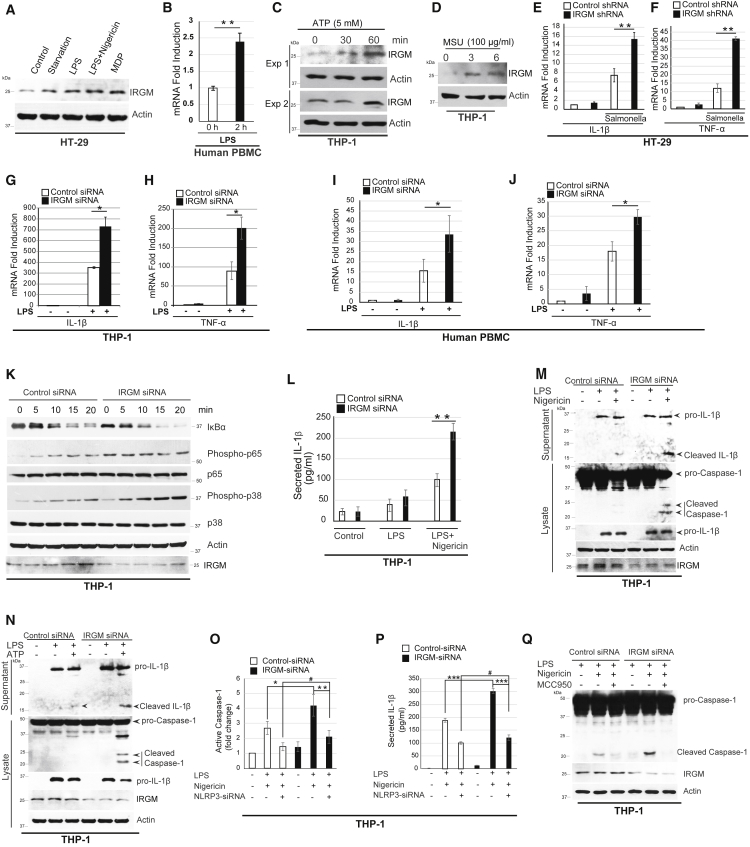
IRGM Suppresses Pro-inflammatory Response and NLRP3-Inflammasome Activation (A) Human colon epithelial HT-29 cells were starved (2 hr) or stimulated with LPS (100 ng/mL, 2 hr) alone or in combination with nigericin (10 μM, 1 hr) or with MDP (10 μg/mL, 6 hr), and immunoblotting was performed with lysates. (B) Human PBMCs from healthy volunteers were exposed to LPS (100 ng/mL), and total RNA was subjected to qRT-PCR using IRGM TaqMan probe. (C and D) THP-1 cells were stimulated with inflammasome inducers (C) ATP or (D) MSU crystals for the indicated time periods, and extracts were subjected to western blotting with IRGM antibody. (E and F) HT-29 control and IRGM knockdown cells were infected with *S. typhimurium* (1:10 MOI, 8 hr), and the total RNA was subjected to qRT-PCR with (E) IL-1β and (F) TNF-α. (G–J) The total RNA isolated from the LPS-stimulated (100 ng/mL, 2 hr) control and IRGM siRNA-transfected (G and H) THP-1 cells or (I and J) PBMCs from five healthy donors were subjected to qRT-PCR for the indicated genes. For (G) and (H), n = 3, mean ± SE, ^∗^p < 0.05, Student’s unpaired t test. For (I) and (J), n = 5, mean ± SE, ^∗^p < 0.05, Student’s paired t test. (K) The LPS (500 ng/mL)-stimulated control and IRGM siRNA-transfected THP-1 cell lysates were subjected to immunoblotting with indicated antibodies. (L) The supernatants from control and IRGM siRNA-transfected THP-1 cells, which were stimulated with LPS (100 ng/mL, 4 hr) alone or in combination with nigericin (5 μM, 30 min), were subjected to ELISA with IL-1β antibody. (M and N) The western blotting was performed with control and IRGM siRNA-transfected THP-1 cells, which were stimulated with LPS (1 μg/mL for 3 hr) alone or in combination (M) with nigericin (5 μM, 30 min) or (N) with ATP (2.5 mM, 4 hr). (O and P) Quantification of (O) active caspase-1 (FLICA assay) and (P) secreted IL-1β (ELISA) in THP-1 cells transfected with control, IRGM, and NLRP3 siRNA and treated with LPS (1 μg/mL, 3 hr) and nigericin (5 μM, 15 min). (Q) The control and IRGM siRNA-transfected THP-1 cells were treated with LPS (1 μg/mL, 3 hr), nigericin (5 μM, 15 min), or MCC950 (1 μM) as indicated, and western blotting was performed. Unless otherwise stated above, n = 3, mean ± SD, ^∗^p < 0.05, ^∗∗^p < 0.005, ^∗∗∗^p < 0.0005, ^#^insignificant, Student’s unpaired t test. See also Figure S1.

Human *IRGM* is mainly expressed in cells of myeloid and epithelial origin, and this expression is increased following exposure of interferon (IFN)-γ (Chauhan et al., 2015). IRGM expression in the colon epithelial cell line HT-29 is increased under starvation conditions and by treatment of cells with the pathogen-associated-molecular-patterns (PAMPs) such as lipopolysaccharide (LPS) and muramyl dipeptide (MDP) (Figures 1A and S1A). In human peripheral blood mononuclear cells (PBMCs), IRGM expression was increased on treatment with LPS (Figure 1B). Further, the treatment of THP-1 cells with danger-associated molecular patterns (DAMPs) such as ATP, MSU (Monosodium urate), and cholesterol crystals increased protein expression of IRGM (Figures 1C, 1D, and S1B). The expression of IRGM was increased on infection of THP-1 cells with *Salmonella typhimurium* (SL1433) (Figure S1C). Thus, *IRGM* expression is induced by DAMPs, PAMPs, and microbes in innate immune cells.

Next, we investigated the role of IRGM in modulating the pro-inflammatory responses induced by LPS treatment and by Salmonella infection. The control and IRGM stable knockdown HT-29 human colon epithelial cells were infected with *Salmonella typhimurium*, and the expression of sentinel pro-inflammatory genes *Il1b*, *Tnfa*, *Il18*, and *Rantes* were monitored by qRT-PCR. As compared to the control cells, IRGM-depleted cells mounted a stronger pro-inflammatory response (Figures 1E, 1F, S1D, and S1E). Similarly, in THP-1 cells, the LPS induced *Il1b* and *Tnfa*, expression was further increased in IRGM small interfering RNA (siRNA) knockdown cells (Figures 1G and 1H). In these cells, *Il18* expression was increased but not significantly, and *Rantes* expression was not affected (Figures S1F and S1G). Further, human PBMCs from healthy donors showed enhanced production of pro-inflammatory cytokine *Il1b* and *Tnfa* when IRGM was knockdown (Figures 1I and 1J). Furthermore, the secretion of IL-6 and tumor necrosis factor alpha (TNF-α) was significantly higher in IRGM-depleted THP-1 cells as compared to the control cells (Figures S1H and S1I). Taken together, the data suggest that IRGM suppresses the pro-inflammatory cytokine response.

To determine the pro-inflammatory signaling pathway controlled by IRGM, we performed a time-course experiment of LPS treatment in control and IRGM-depleted THP-1 cells. As compared to the control cells, the IRGM-depleted cells have lesser IκBα that was degraded faster with LPS treatment (Figure 1K). In the agreement, phospho-NF-κB-p65 levels were higher in IRGM-depleted cells at all the time points (Figure 1K). A previous study in mice indicated the role of p38 MAPK signaling pathway in Irgm1-mediated LPS (TLR4) induced a pro-inflammatory cytokine response (Bafica et al., 2007). Here, in human cells, we found a similar increase in phospho-p38 in IRGM knockdown cells compared to control cells (Figure 1K) suggesting that human IRGM inhibit both NF-κB and p38 MAPK pro-inflammatory signaling pathways to control pro-inflammatory cytokine response.

We note that our several attempts to generate an IRGM knockout human monocytic cell line or a colon epithelial cell lines by different CRISPR-CAS9 methods were unsuccessful. Consequently, in this study, we used siRNA (THP-1 and PBMCs) or small hairpin RNA (shRNA) (HT-29 cells) to transiently or stably knock down the human IRGM; typical knockdown efficiency in any given cell or cell line (PBMC, THP-1, HT-29) was found to be 40%–60% (Figures S1J–S1L).

### IRGM Limits NLRP3 Inflammasome Activation

IL-1β is translated as pro-IL-1β that stays in inactivated form until a second signal activates inflammasomes leading to pro-IL-1β cleavage by caspase-1 to a mature form that is secreted (Choi and Ryter, 2014). We next investigated whether IRGM controls the levels of secreted IL-1β. In ELISA assays, the secreted IL-1β levels were significantly increased on silencing IRGM (Figure 1L) indicating that IRGM suppresses IL-1β cleavage and secretion. The treatment of THP-1 cells with LPS and the inflammasome inducers nigericin (Figure 1M) or ATP (Figure 1N) resulted in increased cleavage of caspase-1 and IL-1β in IRGM-depleted cells compared to the control cells. The results indicate that IRGM suppresses the cleavage of caspase-1 and IL-1β presumably by suppressing NLRP3 inflammasome. We validated this notion by depleting (by siRNA) and inhibiting (by MCC950) the NLRP3 in IRGM knockdown cells. The enhanced inflammasome activation in IRGM-depleted THP-1 cells was blunted when NLRP3 was depleted or inhibited (Figures 1O–1Q and S1M) suggesting that IRGM inhibits NLRP3 activity to suppress activation of caspase-1 and IL-1β.

### IRGM Interacts and Co-localizes with NLRP3 and ASC over the Inflammasomes

**Figure 2:**
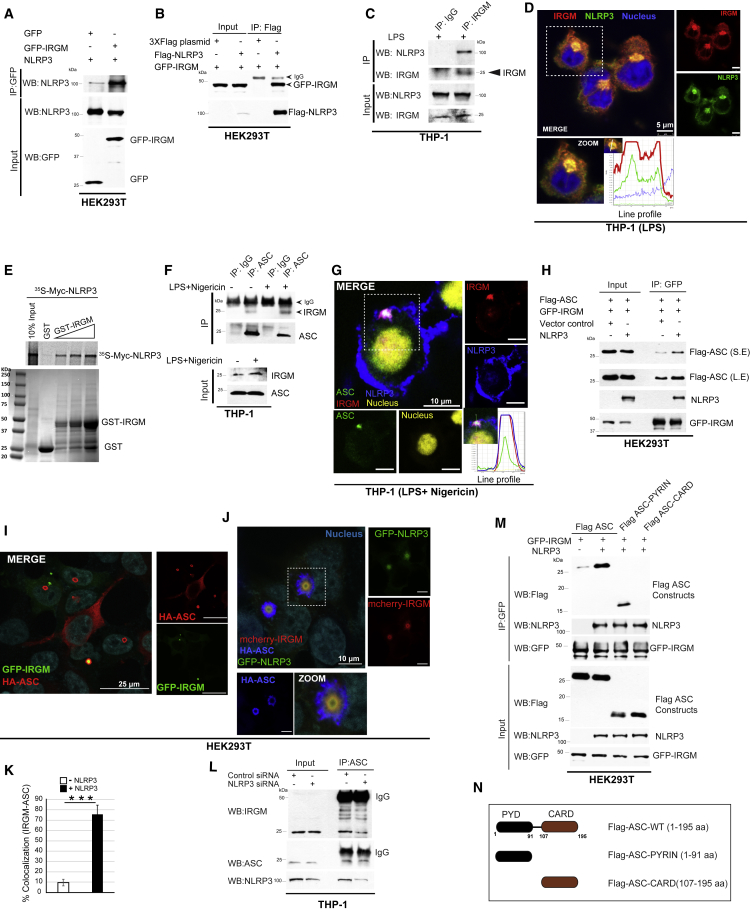
IRGM Interacts and Co-localizes with NLRP3 Inflammasome Components (A and B) Co-immunoprecipitation (coIP) analysis of interaction between (A) GFP-IRGM and NLRP3 or (B) Flag-NLRP3 and GFP-IRGM in HEK293T cells lysates. (C) IP analysis of interaction between endogenous IRGM and NLRP3 in LPS-stimulated THP-1 cells. (D) Representative confocal images of THP-1 macrophages, treated with LPS and processed for immunofluorescence (IF) analysis. (E) GST pull-down assay of in-vitro-translated and radiolabeled myc-tagged NLRP3 with GST or GST-tagged IRGM. (F) IP analysis of interaction between endogenous IRGM and ASC in LPS+nigericin-treated THP-1 cells. (G) Representative confocal images of THP-1 cells, treated with LPS (1 μg/mL, 3 hr) and nigericin (5 μM, 30 min) and processed for IF analysis. (H) CoIP analysis of interaction between IRGM and ASC in HEK293T cell lysates in absence and in presence of NLRP3. S.E., short exposure; L.E., long exposure. (I and J) Representative confocal images of HEK293T cells transiently expressing (I) GFP-IRGM and HA-ASC and (J) GFP-NLRP3, mcherry-IRGM, and HA-ASC. (K) The graph depicts percentage co-localization of ASC specks with IRGM in the absence and presence of NLRP3 (n = 3, mean ± SD, ^∗∗∗^p < 0.0005). (L) IP analysis of the interaction between endogenous IRGM and ASC in THP1 cells transfected with control siRNA or NLRP3 siRNA. (M) CoIP analysis to map the interaction of ASC domains with IRGM in HEK293T cell lysates. (N) The domain organization map of ASC and deletion constructs cloned as FLAG-tagged proteins. Scale bars are indicated in the respective figures. See also Figure S1.

Several diverse signals can activate NLRP3 leading to the oligomerization of NLRP3, the adaptor protein ASC, and the caspase-1 enzyme for the assembly of an activated inflammasome (Choi and Ryter, 2014). Since we found that IRGM suppresses cleavage of caspase-1 and IL-1β, next, we examined the mechanism by which IRGM suppresses the activity of NLRP3 inflammasome. First, we investigated whether IRGM physically associates with inflammasome components. In immunoprecipitation assays, IRGM strongly interacted with both exogenous and endogenous NLRP3 (Figures 2A–2C and S1N). The endogenous IRGM and NLRP3 interacted in THP-1 monocytes (Figure 2C), and the interaction was increased in the presence of *Salmonella typhimurium* (Figure S1N). Endogenous as well as exogenously expressed IRGM was found to be completely co-localized with the NLRP3 in the perinuclear regions of the cells (Figures 2D, S1O, and S1P). In GST pull-down assays, purified IRGM directly interacted with NLRP3 in a concentration-dependent manner (Figure 2E). Taken together, the data suggest that IRGM directly interacts and co-localizes with NLRP3.

On inflammasome activation, ASC oligomerizes with itself and with NLRP3 and caspase-1 to form an active inflammasome complex. In immunoprecipitation assays, performed in THP-1 cells, IRGM interacted with ASC, both under basal and inflammasome-inducing conditions (Figure 2F). ASC formed distinct specks in the cells that are hallmarks of inflammasome activation. Under inflammasome-inducing conditions, IRGM co-localized with ASC and NLRP3 in the core region of specks (Figures 2G, S1Q, and S1R). In co-immunoprecipitation (coIP) experiments, IRGM interacted with ASC, and this interaction was increased when NLRP3 was co-expressed (Figure 2H). In agreement with this, the co-localization of overexpressed ASC structures and IRGM was dramatically increased when NLRP3 was co-expressed (Figures 2I–2K). Also, the depletion of NLRP3 reduced the ASC-IRGM interaction (Figure 2L). The data show that NLRP3 is important for bridging IRGM and ASC. Next, in a domain-mapping experiment, we found that IRGM interacts with PYRIN domain but not with the CARD domain of ASC (Figures 2M and 2N). The PYRIN is the oligomerization domain of ASC. Taken together, the data suggest that IRGM is part of NLRP3 inflammasome where it complexes with both NLRP3 and ASC.

### IRGM Interferes with NLRP3 Inflammasome Assembly

**Figure 3:**
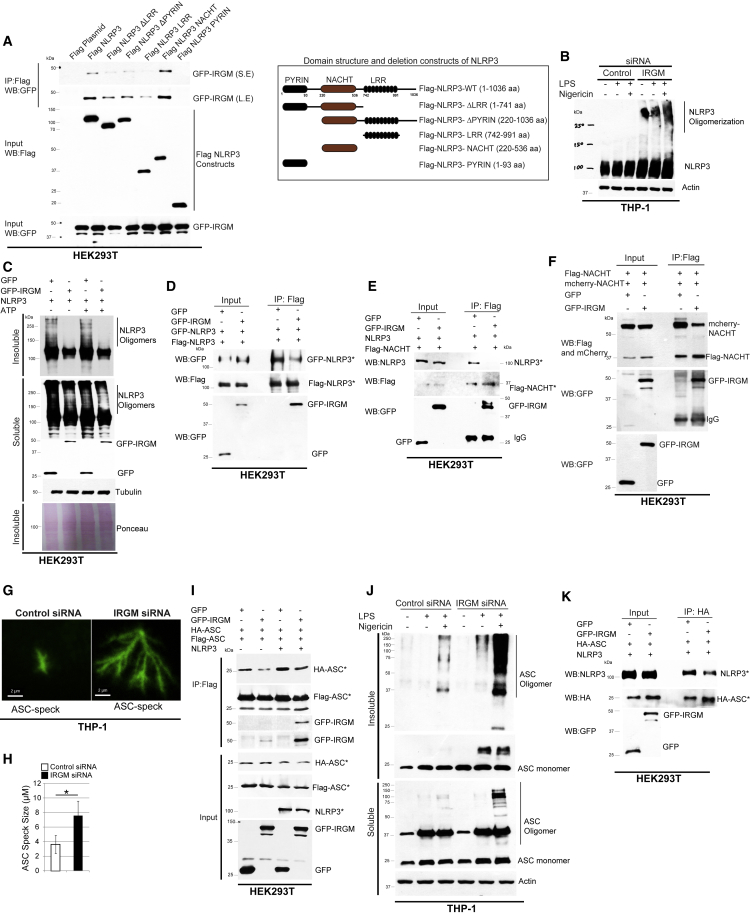
IRGM Impedes the Homo- and Hetero-oligomerization of NLRP3 and ASC (A) Left panel, coIP analysis to map the binding of IRGM over the NLRP3 domains in HEK293T cell lysates. Right panel, the domain organization map of NLRP3 and deletion constructs cloned as FLAG-tagged proteins. S.E., short exposure; L.E., long exposure. (B) The western blotting analysis of DSS cross-linked insoluble fraction of LPS and nigericin-treated control and IRGM knockdown THP-1 cells. (C) The soluble and insoluble fractions of DSS cross-linked HEK293T cells lysates expressing NLRP3 and GFP or GFP-IRGM were subjected to western blotting. (D–F) CoIP analysis of interaction between (D) FLAG-NLRP3 and GFP-NLRP3, (E) FLAG-NLRP3-NACHT and NLRP3, and (F) FLAG-NLRP3-NACHT and mcherrry-NLRP3-NACHT in the absence and presence of GFP-IRGM in HEK293T cells lysates. (G) Super-resolution micrograph of control and IRGM siRNA-transfected THP-1 cells stably expressing LPS-inducible GFP-ASC. (H) Average ASC speck size measured (n = 2, mean ± SD [40 specks], ^∗^p < 0.05). (I) IP analysis from HEK293T cell lysates expressing HA-ASC, FLAG-ASC, and NLRP3 along with GFP control vector or GFP-IRGM vector. (J) The western blot analysis with DSS cross-linked insoluble fraction and soluble fractions of control and IRGM knockdown THP-1 cells stimulated with LPS (1 μg/mL, 3 hr) and nigericin (5 μM, 15 min). (K) IP analysis of interaction between NLRP3 and ASC in the absence and presence of GFP-IRGM. ^∗^In order to reduce the artifact coming from IRGM-mediated degradation of NLRP3 and ASC, the inputs ratios were adjusted to have equal inputs in both the conditions and the IP samples were run in the same ratios as of the inputs. Scale bars are as indicated in figures. See also Figure S2.

To further understand the details of the NLRP3-IRGM interaction, we mapped the binding of IRGM to functional domains in NLRP3 (Figure 3A). NLRP3 is composed of PYRIN, NACHT (nucleotide binding domain or NBD), and LRR domains (Figure 3A). We found that IRGM predominantly interacted with the NACHT domain (Figure 3A). A weak interaction with LRR and almost negligible interaction with PYRIN domain were observed (Figure 3A). The NACHT domain of NLRP3 is an ATP binding domain with an ATPase activity (Duncan et al., 2007). An intact NACHT domain of NLRP3 is required for its oligomerization and activation (Duncan et al., 2007). Hence, we examined whether IRGM perturbs the oligomerization of NLRP3. Indeed, in crosslinking experiments, IRGM depletion was sufficient to increase oligomerization of NLRP3 (Figure 3B). In contrast, the oligomeric forms of overexpressed NLRP3 were reduced when IRGM was overexpressed (Figure 3C). Further, in coIP assays, IRGM was able to reduce homotypic interaction between FLAG-NLRP3 and GFP-NLRP3 (Figure 3D). Furthermore, overexpression of IRGM reduced the interaction between full-length NLRP3 and NACHT domain (Figure 3E) and also between FLAG-NACHT and mCherry-NACHT suggesting that IRGM disrupts the homo-oligomerization of the NLRP3 (Figure 3F). In immunofluorescence assays, the size of NLRP3 aggregates was reduced in the presence of IRGM (Figures S2A–S2C). Altogether, the data show that IRGM restricts the oligomerization of NLRP3.

ASC possess prion-like properties of self-association and forms branched fiber-like structure important for inflammasome activation (Cai et al., 2014). A high-resolution microscopy of ASC specks in THP-1 cells revealed a significantly larger and branched ASC specks in IRGM knockdown cells compared to the control cells (Figures 3G and 3H). In contrast, when IRGM was overexpressed, ASC specks were found to be significantly smaller in overexpressing cells compared to the control cells (Figures S2D–2F). The data indicate that IRGM suppresses the polymerization of ASC. To corroborate these findings, we performed biochemical assays. In coIP experiments, the overexpression of IRGM reduced interaction between HA-ASC and FLAG-ASC (Figure 3I). In western blotting experiments performed with cross-linked insoluble cellular fraction, ASC oligomerization was inhibited by overexpression of IRGM (Figure S2G). In contrast, the amount of oligomerized ASC in the insoluble fraction of the THP-1 cells was markedly increased in IRGM knockdown cells compared to the control cells (Figure 3J). Taken together, these results suggest that IRGM obstructs the oligomerization of ASC also. The interaction between NLRP3 and ASC was also reduced in the presence of IRGM (Figure 3K, see also Figure 5E), but the interaction between ASC and caspase-1 was unaffected by IRGM (Figure S2H). Overall, we found that IRGM restricts assembly of the inflammasome by impeding the homo- and hetero-oligomerization of NLRP3 and ASC.

### IRGM Mediates Autophagic Degradation of NLRP3 Inflammasome Components

**Figure 4:**
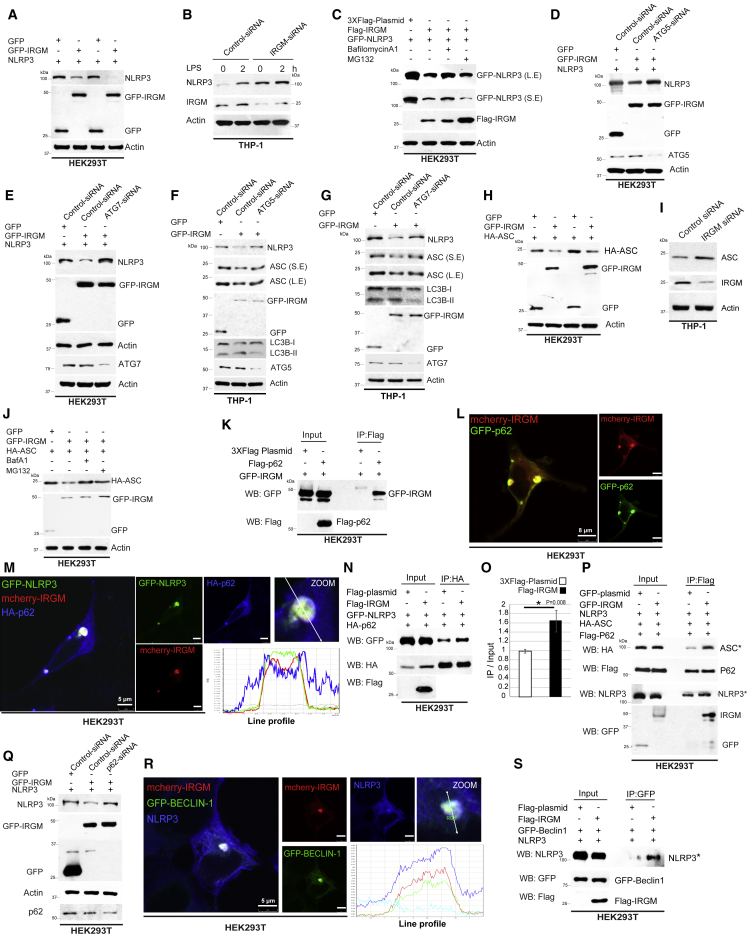
IRGM Mediates Autophagic Degradation of NLRP3 and ASC (A) Western blotting experiment with HEK293T cells expressing NLRP3 and GFP or GFP-IRGM plasmids. (B) Western blot analysis with untreated or LPS-treated (100 ng/mL, 2 hr) control or IRGM siRNA-transfected THP-1 cells lysates. (C) HEK293T cells expressing FLAG control vector, FLAG-IRGM, and GFP-NLRP3 were treated with MG132 or Bafilomycin A1, and lysates were subjected to western blotting. S.E., short exposure; L.E., long exposure. (D and E) The control and (D) ATG5 siRNA or (E) ATG7 siRNA-transfected HEK293T cells expressing NLRP3, GFP, or/and GFP-IRGM were subjected to immunoblotting. (F and G) The control and the (F) ATG5 siRNA or (G) ATG7 siRNA-transfected THP-1 cells expressing GFP or GFP-IRGM were stimulated with LPS (1 μg/mL, 3 hr), and the cell lysates were subjected to immunoblotting. (H) HEK293T cells expressing HA-ASC and GFP or GFP-IRGM were subjected to immunoblotting. (I) The control and IRGM siRNA-transfected THP-1 cells lysates were subjected to immunoblotting. (J) Western blotting analysis with lysates from HEK293T cells transfected with HA-ASC and GFP or GFP-IRGM, untreated or treated with BafA1 or MG132. (K) CoIP analysis of interaction between FLAG-p62 and GFP-IRGM in HEK293T cell lysates. (L and M) Representative confocal images of HEK293T cells expressing (L) GFP-p62 and mcherry-IRGM (M) HA-p62, mcherry-IRGM, and GFP-NLRP3. (N) CoIP analysis of interaction between HA-p62 and GFP-NLRP3 in the absence and presence of FLAG-IRGM. (O) Graph depicts the quantification of GFP-NLRP3 band intensity in IP (normalized compared to inputs) (n = 3, mean ± SD, ^∗^p ≤ 0.05, Student’s unpaired t test) (P) CoIP analysis of interaction between p62 and ASC in the absence and presence of IRGM in HEK293T cell. (Q) Analysis of degradation of NLRP3 in the absence and presence of IRGM and in HEK293T cells transfected with control siRNA and p62 siRNA. (R) Representative confocal images of HEK293T cells expressing mcherry-IRGM, GFP-Beclin-1, and NLRP3. (S) CoIP analysis of interaction between NLRP3 and Beclin-1 in the absence and presence of IRGM in HEK293T cells expressing the indicated plasmids. ^∗^In order to reduce the artifact coming from IRGM-mediated degradation of NLRP3 and ASC, the inputs ratios were adjusted to have equal inputs in both the conditions, and the IP samples was run in the same ratios as of the inputs. Scale bars are as indicated in figures. S.E., short exposure; L.E., long exposure. See also Figures S2 and S3.

We found that the overexpression of IRGM reduced NLRP3 levels (Figure 4A, S2I, and S2J), while silencing IRGM by siRNA increased the total amount of endogenous NLRP3 (Figures 4B and S2K). The data indicate that IRGM mediates degradation of the NLRP3 protein.

NLRP3 is known to be degraded by both the proteasome and autophagy (Kimura et al., 2015, Song et al., 2016). The Bafilomycin A1 (BafA1, an autophagy inhibitor), but not MG132 (a proteasome inhibitor), was able to restore the IRGM-mediated degradation of NLRP3 indicating a role for autophagy in the degradation process (Figures 4C and S2L). In the absence of the essential autophagy proteins, ATG5 or ATG7, IRGM was not able to degrade NLRP3 (Figures 4D–4G and S2M) suggesting that IRGM degrades NLRP3 via autophagy process.

ASC as a part of an inflammasome was previously shown to be degraded by autophagy (Shi et al., 2012). Here, we found that overexpression of IRGM reduced ASC protein levels (Figures 4H and S2N), while IRGM depletion resulted in increased ASC amounts (Figures 4I and S2O). This finding was further supported by immunofluorescence experiments showing that the LPS and nigericin-induced ASC specks (inflammasomes) were significantly increased in number on knocking down IRGM (Figures S2P and S2Q). In contrast, overexpression of IRGM significantly reduced the total number of ASC specks (Figures S2R and S2S). These data suggest that IRGM mediates degradation of ASC. This degradation was completely restored in the presence of BafA1, but only partly in the presence of MG132 (Figures 4J and S2T). In the absence of ATG5 or ATG7, IRGM was not able to degrade ASC (Figures 4F and 4G) suggesting that IRGM degrades ASC via autophagy process.

### IRGM Mediates p62-Dependent Selective Autophagy of NLRP3 Inflammasomes

p62 is a selective autophagy adaptor protein that plays a vital role in autophagic degradation of several inflammatory signaling pathway proteins including inflammasomes and their components (Chen et al., 2016, Samie et al., 2018, Shi et al., 2012). Here, we explored whether IRGM acts as a scaffold protein to mediate p62-dependent selective autophagy of NLRP3. We found that IRGM interacted and co-localized with p62 (Figures 4K and 4L). IRGM was also required for expression of p62 under basal and inflammasome-inducing conditions in THP-1 cells (Figure S3A). NLRP3 itself can interact and co-localize with p62 (Figures S3B–3D). In THP-1 cells, p62 was co-localized with NLRP3 (Figure S3D) on perinuclear structures that appeared similar to those on which IRGM and NLRP3 co-localized (Figure 2D) suggesting that all the three proteins are part of the same complex. In HEK293T cells, overexpressed IRGM, p62, and NLRP3 co-localized with each other suggesting that they are indeed in a single complex (Figure 4M). IRGM increased the interaction of p62 with both NLRP3 (Figures 4N and 4O) and ASC (Figure 4P) suggesting that IRGM is vital for bridging p62 with NLRP3- and ASC-containing inflammasomes.

Next, we asked whether p62 as a part of the IRGM-p62-NLRP3 complex is required for IRGM-mediated autophagic degradation of NLRP3. Indeed, depletion of p62 by siRNA restored IRGM-mediated NLRP3 degradation (Figures 4Q and S3E). ULK1 plays an important role in autophagy initiation, whereas Beclin1 and ATG16L1 are required for autophagosome assembly and elongation. Since IRGM interacts with ULK1, Beclin 1, and ATG16L1 for assembly of the autophagy machinery (Chauhan et al., 2015), we next asked which of these autophagy proteins are utilized by IRGM for autophagy of NLRP3. NLRP3 and IRGM co-localized with all three autophagy regulatory proteins (Figures 4R, S3F, and S3G). Further, in coIP experiments, IRGM robustly increased NLRP3-Beclin1 and NLRP3-ATG16L1 interactions (Figures 4S and S3H) but did not affect NLRP3-ULK1 interaction (Figure S3I). Taken together, the data suggested a scaffolding role for IRGM in bringing the autophagy adaptor protein (p62), autophagy initiation (Beclin 1), and elongation protein (ATG16L1) over the NLRP3 inflammasomes.

We performed time-chase immunofluorescence experiments to understand whether the p62 docks to NLRP3/ASC complex first or the Beclin1/ATG16L1 reaches the complex first. The IRGM-p62-ASC showed co-localization in 5 min of stimulation, whereas ATG16L1 came later at 15 min or more prominently on 30 min post stimulation (Figures S4A and S4B). We also performed time-chase immunofluorescence experiments in overexpression system in HEK293T cells. The data show that, in the presence of p62, the NLRP3-IRGM puncta formation are fast, and all three p62, IRGM, and NLRP3 co-localized with each other 6 hr post-transfection (Figures S4C and S4D), whereas in the presence of Beclin1 the IRGM-NLRP3 complex appeared at 12 hr post-transfection (Figures S4C and S4D). Taken together, the data suggest that p62 being an adaptor protein recognizes the inflammasome first followed by docking of other autophagy proteins.

**Figure 5:**
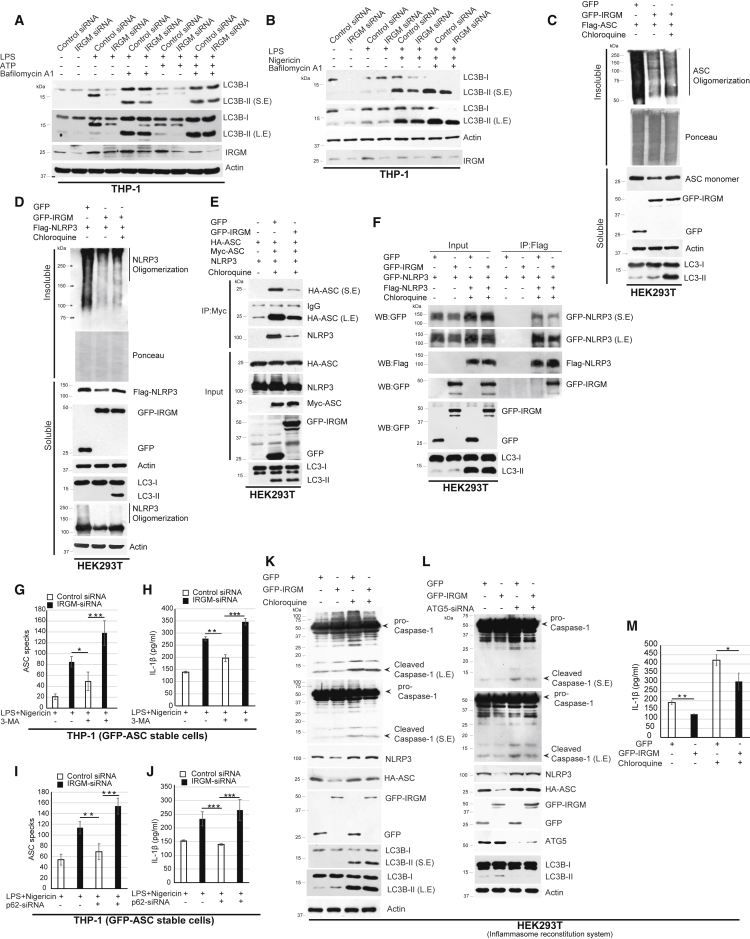
Both Autophagy and Inflammasome Assembly Defect Triggered by IRGM Leads to Inhibition of NLRP3 Inflammasome Activation (A and B) Analysis of regulation of autophagy flux in control and IRGM knockdown THP-1 cells stimulated with LPS (1 μg/mL, 3 hr) or (A) LPS +ATP (5 mM, 60 min) or (B) LPS+nigericin (5 μM, 15 min) with or without Bafilomycin A1 (300 nM, 3 hr). (C and D) Western blotting analysis of the DSS cross-linked insoluble and soluble fraction of HEK293T cells expressing (C) FLAG-ASC or (D) FLAG-NLRP3 and GFP or GFP-IRGM in absence or presence of chloroquine (50 μM, 5 hr). (E and F) CoIP analysis of interaction between (E) myc-ASC and HA-ASC or (F) GFP-NLRP3 and FLAG-NLRP3 in the absence and presence of GFP-IRGM in HEK293T cells treated or untreated with chloroquine (50 μM, 5 hr). (G–J) Average number of (G and I) ASC specks or (H and J) secreted IL-1 β from THP-1 GFP-ASC stable cells transfected with control and IRGM siRNA and stimulated with LPS+nigericin, in the absence and presence of (G and H) 3-MA (10 mM, 3 hr) or (I and J) p62 siRNA (n = 3, mean ± SD, ^∗^p ≤ 0.05, ^∗∗^p ≤ 0.005, ^∗∗∗^p ≤ 0.0005, Student’s unpaired t test). (K–M) HEK293T cells transfected with NLRP3, HA-ASC, caspase-1, pro-IL-1β, GFP-IRGM, and GFP for 30 hr and (K and M) treated with chloroquine for next 6 hr or (L) transfected with ATG5 siRNA. (K and L) The cell lysates were subjected to western blotting and (M) supernatant used in ELISA to measure IL-1β. (n = 3, mean ± SD, ^∗^p ≤ 0.05, ^∗∗^p ≤ 0.005, Student’s unpaired t test). S.E., short exposure; L.E., long exposure. See also Figures S4 and S5.

NLRC4 and AIM2 are other well-studied inflammasomes. The NLRC4 levels were unchanged, whereas, surprisingly, AIM2 levels were decreased on knocking down IRGM (Figures 5A and 5B). However, the overexpression of IRGM did not significantly affect the levels of overexpressed AIM2 in HEK293T (Figures S5C and S5D) suggesting that the reduction in AIM2 expression observed in IRGM knockdown THP-1 cells is not a direct effect of IRGM low expression but could be a secondary event. This notion is supported by negligible AIM2-IRGM co-localization (Figures S5E and S5F). Taken together, our data show that AIM2 or NLRC4 are not degraded by IRGM-mediated selective autophagy, and the discovered mechanism is specific to NLRP3.

Next, we investigated whether the LPS and inflammasome activator-induced general autophagy flux is controlled by IRGM. The results suggest that LPS-, LPS+nigericin-, and LPS+ATP-induced autophagy flux is inhibited upon IRGM depletion (Figures 5A and 5B), whereas the LPS+poly(dA-dT) (an AIM2 inflammasome inducer)-induced autophagy flux was not affected by knockdown of IRGM (Figure S5G).

IRGM mediates selective autophagy of NLRP3 and ASC and also regulates basal autophagy. We asked whether IRGM itself is an autophagy target. In cycloheximide chase experiments, we found that MG132 but not BafA1 could protect IRGM from degradation suggesting that IRGM is degraded by proteasome but not by the autophagy (Figures S5H and S5I).

### Two Distinct Mechanisms Governed by IRGM Regulate NLRP3 Inflammasome Suppression

IRGM utilizes two mechanisms: (1) suppression of oligomerization and (2) autophagic degradation of NLRP3/ASC to inhibit the inflammasome activation. Next, we performed experiments to determine whether these two mechanisms are distinct or dependent on each other. In oligomerization assays, in the presence of autophagy inhibitor chloroquine, although the IRGM-mediated degradation of NLRP3 and ASC monomers (soluble fraction) was significantly restored, the oligomerization defect of NLRP3 and ASC was not rescued (Figures 5C and 5D). These data indicate that the IRGM-mediated autophagic degradation of NLRP3 and ASC is independent of IRGM-regulated inhibition of oligomerization. Next, we found that IRGM was able to inhibit the oligomerization of NLRP3, as well as ASC even in the presence of autophagy inhibitor (Figures 5E and 5F) suggesting that IRGM-mediated oligomerization defect is independent of IRGM-controlled autophagic degradation of NLRP3 and ASC.

A recent study showed that a single mutation in the GTPase domain (S47N) of IRGM makes it incompetent in interaction with Syntaxin 17, an autophagosome-lysosome fusion protein (Kumar et al., 2018). We found that this mutation (S47N, IRGM GTPase activity defective) in IRGM renders it inefficient in degrading NLRP3 suggesting the importance of IRGM GTPase activity in autophagy-mediated cargo degradation (Figure S5J). Very interestingly, this mutation was able to rescue the IRGM-mediated autophagic degradation of NLRP3 but not the oligomerization defect mediated by IRGM (Figure S5J). Taken together, the results suggest that IRGM controls two distinct mechanisms, autophagic degradation of NLRP3 (GTPase-dependent function) and inhibition of oligomerization of NLRP3 (GTPase-independent function).

Next, we performed several experiments to parse out the relative contribution of these two mechanisms in inhibiting NLRP3 inflammasome activation. We observed that the treatment of 3-MA (an autophagy inhibitor) results in increased inflammasomes (ASC specks) numbers and IL-1β secretion compared to the untreated THP-1 cells suggesting that autophagy reduces the NLRP3 inflammasome formation and activation (Figures 5G and 5H and S5K). We found that knockdown of IRGM induces significantly more ASC specks formation and also IL-1β secretion as compared to the 3-MA-treated THP-1 cells (Figures 5G and 5H). These data indicate that IRGM-mediated suppression of inflammasomes includes mechanisms more than autophagy-mediated degradation. The 3-MA-treated IRGM knockdown cells showed significantly higher ASC speck formation and IL-1β secretion than 3-MA-treated control cells (Figures 5G and 5H) suggesting that, in addition to autophagy-mediated suppression, the IRGM-mediated inhibition of oligomerization of inflammasomes may play a significant role in suppressing NLRP3 inflammasomes. Similar results were obtained when selective autophagic degradation of inflammasome was inhibited by knocking down of p62 in IRGM-depleted THP-1 cells (Figures 5I and 5J). Next, in HEK293T inflammasome reconstitution system, the overexpression of IRGM reduces caspase-1 cleavage (Figures 5K and 5L cf. lane 1 and 2 in SE) and also the IL-1β secretion (Figure 5M). The inhibition of autophagy (using chloroquine or ATG5 knockdown) in IRGM-overexpressing cells increased the caspase-1 cleavage and also IL-1β secretion but to lesser extent than autophagy-inhibited control cells (Figures 5K and 5L, cf. lane 4 with 3 in long exposure [L.E.], Figure 5M) suggesting yet again that IRGM mediates suppression of NLRP3 inflammasome by at least two distinct mechanisms discovered here.

### IRGM Protects from Pyroptosis

**Figure 6:**
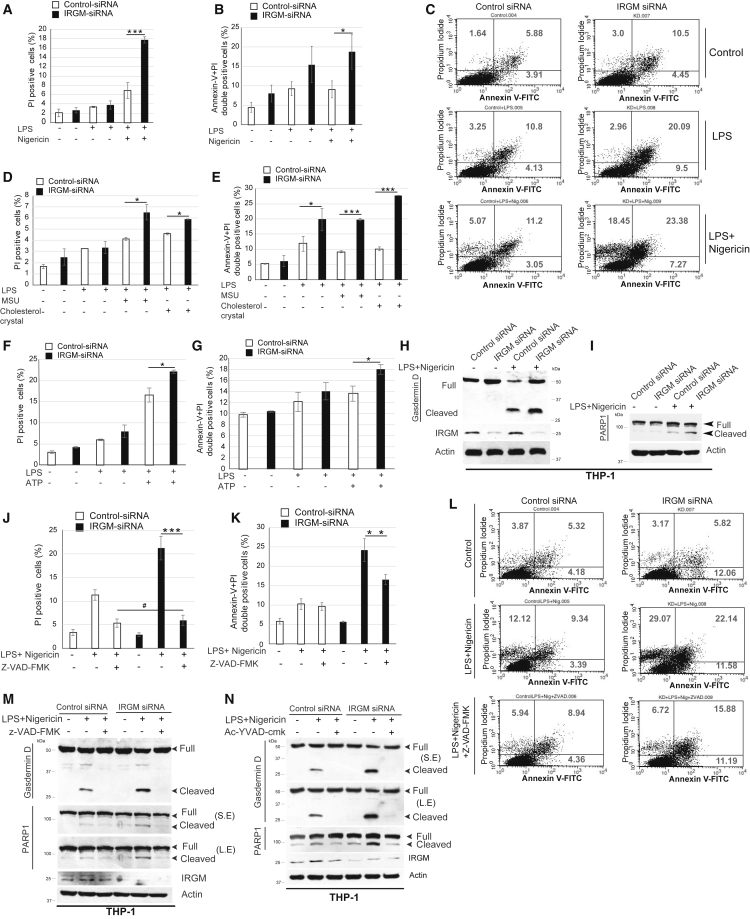
IRGM Protects from Caspase-Dependent Inflammatory Cell Death (A and B) Flow cytometry analysis of control and IRGM siRNA knockdown cells untreated or treated with LPS (1 μg/mL, 3 hr) and nigericin (5 μM, 30 min). Bar graphs show percentage of (A) PI positive or (B) Annexin V/PI double-positive cells. (C) Representative dot plot showing flow cytometry analysis of control and IRGM siRNA knockdown cells untreated or treated with LPS and nigericin. (D–G) Flow cytometry analysis of control and IRGM siRNA knockdown cells untreated or treated with LPS, MSU, cholesterol crystal, and ATP as indicated. Bar graph showing percentage of (D and F) PI positive and (E and G) Annexin V/PI double-positive cells. (H and I) The control and IRGM siRNA-transfected THP-1 cells treated with LPS and nigericin were subjected to immunoblot analysis with (H) Gasdermin D or (I) PARP1 and actin antibodies. (J and K) Flow cytometry analysis of control and IRGM siRNA knockdown cells untreated or treated with LPS, nigericin, and Z-VAD-FMK (5 μM, 30 min) as indicated. Bar graph showing the percentage of (J) PI positive and (K) Annexin V/PI double-positive cells. (L) Representative dot plot is showing flow cytometry analysis of control and IRGM siRNA knockdown cells untreated or treated with LPS and nigericin and Z-VAD FMK as depicted. (M and N) Western blot analysis of control and IRGM knockdown THP-1 cells untreated or treated with LPS, nigericin, and (M) Z-VAD-FMK or (N) Ac-YVAD-cmk. Unless otherwise stated, n = 3, mean ± SD, ^∗^p ≤ 0.05^∗∗^p ≤ 0.005, ^∗∗∗^p ≤ 0.0005, ^#^insignificant, Student’s unpaired t test). S.E., short exposure; L.E., long exposure. See also Figure S6.

Pyroptosis is an inflammatory cell death mediated by activation of inflammasomes (also called pyroptosomes) (Fernandes-Alnemri et al., 2007, Shi et al., 2017). Annexin V and propidium iodide (PI) double staining was used to access the role of IRGM in pyroptosis. Due to the formation of pores, pyroptotic cells are mainly stained by PI (PI is cell membrane impermeant). Annexin-V cannot distinguish between apoptotic cells and pyroptotic cells and stains both of them. In flow cytometry experiments, on exposure of inflammatory threats, both the apoptotic and pyroptotic populations were increased considerably in IRGM-depleted THP-1 cells compared to control cells (Figures 6A–6G, S6A, and S6B). These results suggest that IRGM protects the cells from inflammation-induced cell death. In the lactate dehydrogenase (LDH) release assays, there was a significant increase in the LDH amount in supernatant collected from LPS- and DAMP-treated IRGM-depleted THP-1 cells compared to the control cells (Figure S6C). Similar results were obtained using a trypan blue exclusion assay that accesses the integrity of the cell membrane during cell death (Figure S6D).

Recent studies identified Gasdermin D (GSDMD) as an effector protein for pyroptosis (Liu et al., 2016, Shi et al., 2015). In agreement with caspase-1 cleavage data presented in Figure 1, the cleavage of GSDMD was increased in IRGM-depleted cells compared to the control cells (Figure 6H). In agreement with flow cytometry data, apoptosis was also increased in IRGM knockdown cells compared to control cells as clear from the enhanced PARP1 (Poly [ADP-ribose] polymerase 1) cleavage (Figure 6I). Taken together, our data show that IRGM protects cells from inflammation-induced cell death.

Next, we blocked the caspase’s activity using the pan-caspase inhibitor z-VAD-FMK or selective caspase-1 inhibitor Ac-YVAD-CMK to determine whether the inflammatory cell death observed in IRGM-depleted cells is caspase dependent or independent. The results (both by flow cytometry and western blotting) show that z-VAD-FMK and Ac-YVAD-CMK can block pyroptosis and also can reduce apoptosis induced by IRGM depletion (Figures 6J–6N) suggesting that IRGM suppresses caspase-1-dependent inflammatory cell death.

### Mouse Irgm1 Suppresses the Gut Inflammation by Inhibiting the NLRP3 Inflammasome Activation

**Figure 7:**
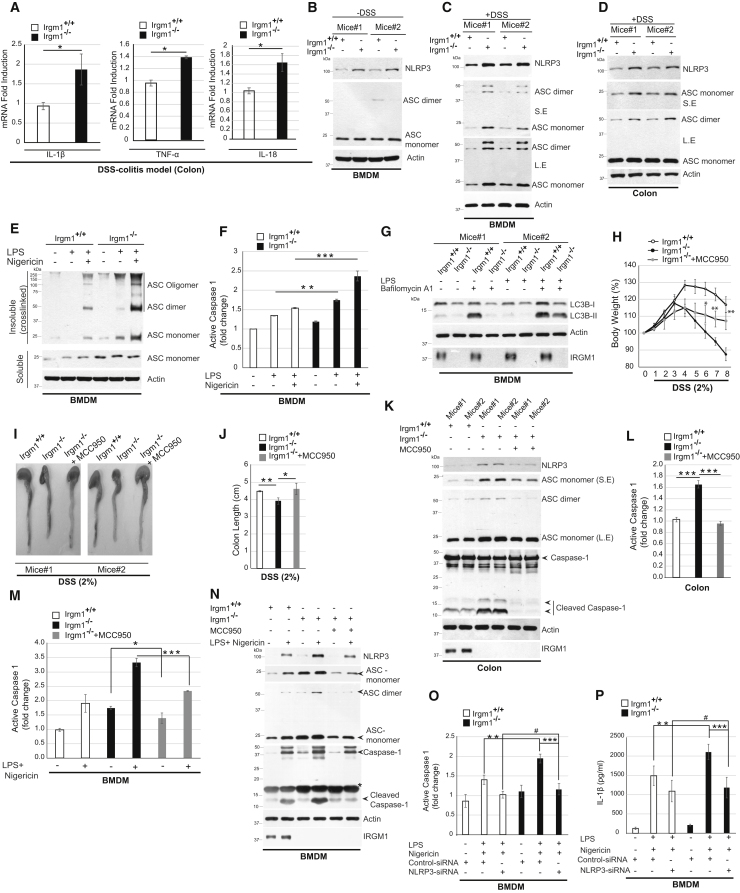
Irgm1 Suppresses Colitis via Inhibition of NLRP3 Inflammasome (A) The qPCR analysis from the total RNA isolated from colon of DSS-treated *Irgm1*^*+/+*^ and *Irgm1*^*−/−*^ knockout mice (n = 3, mean ± SE, ^∗^p < 0.05, Student’s unpaired t test). (B and C) Western blot analysis with BMDM lysates from (B) untreated or (C) DSS-treated *Irgm1*^*+/+*^ and *Irgm1*^*−/−*^ knockout mice with indicated antibodies. (D) Western blot analysis with colon lysates from DSS-treated *Irgm1*^*+/+*^ and *Irgm1*^*−/−*^ mice. (E) Western blotting analysis of cross-linked insoluble and soluble cell fraction from LPS- and nigericin-treated *Irgm1*^*+/+*^ and *Irgm1*^*−/−*^ BMDMs. (F) The quantification of activated caspase-1 (FLICA assay) in LPS- and nigericin-treated *Irgm1*^*+/+*^ and *Irgm1*^*−/−*^ BMDM lysates. (G) Western blotting analysis from LPS- and Bafilomycin A1-treated *Irgm1*^*+/+*^ and *Irgm1*^*−/−*^ BMDM cell lysates. (H) Graph depicting the percentage of change in body weight during the course of DSS treatment. (I) Representative pictures of colons of DSS- and MCC950-treated and -untreated *Irgm1*^*+/+*^ and *Irgm1*^*−/−*^ mice. (J) Graph depicts colon length of DSS- and MCC950-treated and -untreated *Irgm1*^*+/+*^ and *Irgm1*^*−/−*^ mice. (K) Western blot analysis from colon lysates of DSS- and MCC950-treated and -untreated *Irgm1*^*+/+*^ and *Irgm1*^*−/−*^ mice. (L and M) The quantification of activated caspase-1 (FLICA assay) in (L) colon lysates of DSS and MCC950 or (M) BMDM lysates of LPS, nigericin, and MCC950 (1 μM)-treated and -untreated *Irgm1*^*+/+*^ and *Irgm1*^*−/−*^ mice. (N) Western blot analysis from of LPS, nigericin, and MCC950 (1 μM)-treated or -untreated BMDM cell lysates from *Irgm1*^*+/+*^ and *Irgm1*^*−/−*^ mice. ^∗^Non-specific. (O and P) Quantification of active caspase-1 (caspase-1 FLICA assay) (O) and secreted IL-1β (ELISA) (P) in LPS- (100 ng/mL, 3 hr) and nigericin- (5 μM, 15 min) treated NLRP3-depleted BMDMs from *Irgm1*^*+/+*^ and *Irgm1*^*−/−*^ mice. S.E., short exposure; L.E., long exposure. n = 3, mean ± SD, ^∗^p < 0.05, ^∗∗^p < 0.005, ^∗∗∗^p < 0.0005, ^#^insignificant Student’s t test unpaired). See also Figure S7.

Although human IRGM and mouse Irgm1 possess biochemical differences, they have been found to have overlapping functions, particularly as related to innate immunity and autophagy. Given the role of IRGM in inflammatory bowel disease (IBD) and Crohn’s, we utilized the dextran-sulfate (DSS)-induced colitis model to investigate the role of Irgm1 in Nlrp3 inflammasome activation. *Irgm1*^*−/−*^ mice have previously been shown to exhibit enhanced inflammation following DSS administration (Liu et al., 2013). Consistent with this report, we found here that the administration of DSS led to decreased body weight and colon length in *Irgm1*^*−/−*^ mice relative to those responses in littermate *Irgm1*^*+/+*^ mice (Figures S7A–S7D). Like in human colon cells, the expression of *IL-1β*, *TNF-α*, and *IL-18* was significantly increased in DSS-treated *Irgm1*^*−/−*^ compared to *Irgm1*^*+/+*^ mice (Figure 7A). Next, we scrutinized the expression of inflammasome components. In untreated mice, Nlrp3 expression was increased in *Irgm1*^*−/−*^ mice compared to *Irgm1*^*+/+*^ mice (Figure 7B); however, expression of ASC was not different (Figure 7B). In DSS-treated mice, the expression of both Nlrp3 and ASC in bone-marrow-derived macrophages and colon was more in *Irgm1*^*−/−*^ mice compared to *Irgm1*^*+/+*^ mice (Figures 7C and 7D). Also, ASC oligomerization was considerably higher in *Irgm1*^*−/−*^ mice compared to *Irgm1*^*+/+*^ mice (Figures 7C and 7D). In a cross-linking experiment with bone marrow-derived macrophages (BMDMs), both ASC and Nlrp3 oligomerization was found to be increased in *Irgm1*^*−/−*^ mice compared *Irgm1*^*+/+*^ mice (Figures 7E and S7E; STAR Methods). Under inflammasome-inducing conditions, the active caspase-1 amount was significantly higher in *Irgm1*^*−/−*^ mice compared to control *Irgm1*^*+/+*^ mice (Figure 7F). Similar to human cell lines studies, the basal autophagy and also the LPS-induced autophagy flux in BMDMs were dependent on the expression of Irgm1 (Figure 7G). Altogether, the data show that Irgm1, like its human ortholog, suppresses the activation of the NLRP3 inflammasome by inhibiting its assembly and by increasing autophagy.

Next, we analyzed whether increased Nlrp3 inflammasome activation is the cause for exacerbated outcomes of DSS-induced colitis in *Irgm1* knockout mice. MCC950 is the most potent and the most specific small-molecule inhibitor of activation of NLRP3 known to date (Coll et al., 2015, Strangward et al., 2018, Yao et al., 2018). We investigated whether selective pharmacologic blockade of the Nlrp3 inflammasome using MCC950 would reduce the colitis symptoms in *Irgm1*^*−/−*^ mice. Indeed, we found that all the intensified outcomes of DSS-induced colitis in the Irgm1 knockout mice were reversed in the presence of MCC950 (Figures 7H–7J, S7F, and S7G). At molecular levels, in colon tissues, the increased amount of inflammasome components (Nlrp3, ASC monomers, and dimers) and also the enhanced inflammasome activity as measured by cleaved caspase-1 by western blot (Figure 7K) and fluorescent caspase-1 cleavage assay (Figure 7L) in *Irgm1*^*−/−*^ mice were reversed by MCC950 treatment. Next, we isolated the BMDMs from these mice and induced the inflammasome (using LPS+nigericin) in the absence and presence of MCC950. Again, the data show that inactivation of Nlrp3 by MCC950 considerably reduced the enhanced inflammasome activity in *Irgm1*^*−/−*^ mice as measured by fluorescent caspase-1 cleavage assay (Figure 7M) and western blotting (Nlrp3, ASC dimer, and caspase-1 cleavage) (Figure 7N). Next, we used a genetic approach to determine whether enhanced inflammasome activation in Irgm1^−/−^ mice BMDMs is because of increased activation of the Nlrp3/ASC inflammasome. In LPS+nigericin-stimulated BMDMs, the increased caspase-1 cleavage (Figures 7O and S7H) and the IL-1β secretion (Figures 7P and S7H) in *Irgm1*^*−/−*^ mice BMDMs was blunted when Nlrp3 was depleted in these cells using siRNA.

Taken together, several lines of evidence suggest that NLRP3 activation is the primary factor behind enhanced inflammasome activity in IRGM-depleted cells, and also the activation of NLRP3 inflammasome is one of the major reasons for exacerbated outcomes of DSS-induced colitis in the *Irgm1* knockout mice. We conclude that the IRGM/Irgm1 suppresses the NLRP3 inflammasome to keep the gut inflammation under check.

## Discussion

In landmark genome-wide association studies conducted by Wellcome Trust Case Control Consortium to understand the genetic determinants of inflammatory diseases, SNPs in the *IRGM* locus were found to be strongly associated with Crohn’s disease (Wellcome Trust Case Control, 2007, Craddock et al., 2010). Later, several studies showed similar genetic linkages of IRGM with Crohn’s disease and other inflammatory and autoimmune diseases in different populations worldwide (Baskaran et al., 2014, Glas et al., 2013, Li et al., 2014, McCarroll et al., 2008, Xia et al., 2017, Yang et al., 2014). Most of these studies suggested a protective role of IRGM against the inflammatory diseases; however, none of the studies revealed the mechanism(s).

This study defined the mechanism by which IRGM/Irgm1 regulates cellular inflammation in immune and gut epithelial cells (Figure S7I). We found here that IRGM suppresses IL-1β production by limiting the activation of the NLRP3 inflammasome (Figure S7I). Remarkably, IRGM performs this function by complexing directly with inflammasome components including NLRP3 and ASC. By binding to the oligomerization domain (NACHT) of NLRP3, it impedes the oligomerization of NLRP3. IRGM also hinders polymerization of ASC protein (Figure S7I), which is a key event for activation of inflammasome (Cai et al., 2014, Lu et al., 2014). Previous studies demonstrated the role of autophagy process in limiting the IL-1β production, and one of the mechanisms was by degrading the inflammasome and its components in a p62-dependent manner (Shi et al., 2012). Here, we found that IRGM is a key player in mediating p62-dependent selective autophagy of NLRP3 and ASC (Figure S7I). Thus, IRGM limits IL-1β production by two mechanisms: (1) by interfering in the assembly of the inflammasome and (2) by mediating autophagy of inflammasome. In the inflammatory bowel disease experimental mice model, Irgm1 suppresses the colitis by inhibiting the NLRP3 inflammasome. Taken together, this work revealed IRGM-mediated anti-inflammatory immune homeostasis mechanism by which IRGM could be protective against inflammatory diseases.

As compared to humans, where IRGM is a lone member of the IRG family, mice have 20 more homologs of Irgm1, presumably providing the compensation when the Irgm1 is knockout in the mice. Although human IRGM and mice Irgm1 are of different size (21 versus 47 kDa), they are strikingly similar in the regulation of autophagy and inflammation.

The dysregulated NLRP3 inflammasome has been linked to the pathogenesis of several inflammatory diseases including gout, type 2 diabetes, cancer, cardiovascular diseases, Alzheimer’s, Parkinson’s, and prion diseases (Alcocer-Gómez and Cordero, 2017, Lee et al., 2013, Song et al., 2017). Also, mutations in the NLRP3 gene have been linked with a range of dominantly inherited auto-inflammatory diseases (Masters et al., 2009). Therefore, it is of high importance to the medical science to understand the mechanisms by which the cell restrains the activation of NLRP3 inflammasome and IL-1β production. For similar reasons, understanding the protective nature of IRGM in human inflammatory diseases is crucial. This study delineates both. The therapeutic strategies to increase IRGM activity or targeting the IRGM-NLRP3 interaction could be useful for treating IRGM- and NLRP3-associated diseases.

## STAR★Methods

### Key Resources Table

**Table:** 

REAGENT or RESOURCE	SOURCE	IDENTIFIER
**Antibodies**		

NLRP3	Adipogen	Cat #: AG-20B-0014-C100
NLRP3	Cell Signaling	Cat #: 13158S
Anti-Caspase-1 (p20) (human), clone Bally-1	Adipogen	Cat #: AG-20B-0048-C100
GFP	Abcam	Cat #: ab290; RRID:AB_303395
Anti-IRGM Antibody (Human)	Abcam	Cat #: Ab69494; RRID:AB_1209373
IRGM Antibody (Rodent specific)	Cell Signaling	Cat #: 14979S
IL-1β (3A6) Mouse mAb	Cell Signaling	Cat #: 12242; RRID:AB_2715503
HA-Tag (C29F4) Rabbit mAb	Cell Signaling	Cat #: 3724S; RRID:AB_1549585
c-Myc	Santa Cruz Biotechnology	Cat #: sc-40; RRID:AB_627268
Atg5 Antibody	Cell Signaling	Cat #: 2630S
Atg7 (D12B11) Rabbit mAb	Cell Signaling	Cat #: 8558S; RRID:AB_10831194
Anti-pro Caspase1+p10+p12 Antibody (EPR16883)	Abcam	Cat #: ab179515
ASC	Santa Cruz Biotechnology	Cat #: sc-22514R
ATG16L	MBL	Cat #: M150-3; RRID:AB_1278758
ATG16L	MBL	Cat #: PM040; RRID:AB_1278757
Beclin-1	Cell Signaling	Cat #: 3738S; RRID:AB_490837
Anti-LC-3B antibody	SIGMA-ALDRICH	Cat #: L7543; RRID:AB_796155
p62	BD Biosciences	Cat #: BD–610832; RID:AB_398151
NF-kb p65	Santa Cruz Biotechnology	Cat #: sc-8008; RRID:AB_628017
p-NFκB p65 (Ser 536)	Santa Cruz Biotechnology	Cat #: sc-33020;RRID:AB_2179018
p-38α	Santa Cruz Biotechnology	Cat #: sc-535; RRID:AB_632138
Phsopho-p38 MAPK (Thr180/Tyr182)	Cell Signaling	Cat #: 9211; RRID:AB_331641
I kappa B alpha (L35A5)	Cell Signaling	Cat #: 4814; RRID:AB_390781
PARP1	Cell Signaling	Cat #: 9542L; RRID:AB_2160739
Gasdermin D	Cell Signaling	Cat #: 96458
NLRC4 (D5Y8E)	Cell Signaling	Cat #: 12421S
AIM2 (D5X7K)	Cell Signaling	Cat #: 12948S
Anti- beta Actin	Abcam	Cat #: ab6276; RRID:AB_2223210
Rabbit polyclonal to beta tublin	Abcam	Cat#: ab6046; RRID:AB_2210370
Monoclonal Anti-Flag M2	SIGMA-ALDRICH	Cat #: F1804: RRID:AB_262044
Goat Anti-Rabbit IgG H& L (HRP)	Abcam	Cat #: Ab 97051; RRID:AB_10679369
Anti-Mouse IgG (H+L) HRP Conjugate	Promega	Cat #: W4021
Anti-Rabbit IgG (H+L) HRP Conjugate	Promega	Cat #: W4011
Mouse Anti-Rabbit HRP	Santa Cruz Biotechnology	Cat #: sc-2357
Mouse TrueBlot ULTRA: Anti mouse IgG HRP	Rockland	Cat #: 18-8817-33
Rabbit TrueBlot ULTRA: Anti Rabbit IgG HRP	Rockland	Cat #: 18-8816-33
Alexa Fluor 488 goat anti-mouse	Invitrogen	Cat #: A11029
Alexa Fluor 488 goat anti-Rabbit	Invitrogen	Cat #: A11034
Alexa Fluor 568 goat anti-mouse	Invitrogen	Cat #: A11031
Alexa Fluor 568 goat anti-Rabbit	Invitrogen	Cat #: A11036
Alexa Fluor 647 goat anti-mouse	Invitrogen	Cat #: A21236
Alexa Fluor 647 goat anti-Rabbit	Invitrogen	Cat #: A21245

**Bacterial and Virus Strains**		

*E.Coli* DH5 alpha	Lab strain	N/A
*Salmonella typhimurium* strain *S*T 1433 strain	Lab strain	N/A

**Biological Samples**		

Mouse colon tissue	C57 BL/6 irgm1^+/+^ and irgm1^−/−^	NA
Human PBMCs	Healthy donors blood	NA

**Chemicals, Peptides, and Recombinant Proteins**		

Lipopolysaccharides from *Escherichia coli* O111:B4	SIGMA-ALDRICH	Cat #: L4391
Lipopolysaccharides (LPS)	CST	Cat #: 14011S
Muramyl Dipeptide, MDP	InvivoGen	Cat #: tlrl-mdp
Poly (I:C) LMW/LyoVec	InvivoGen	Cat #: tlrl-picwlv
Poly (dA:dT)/LyoVec	InvivoGen	Cat #: tlrl-patc
Phorbol 12-myristate 13-acetate, PMA	SIGMA-ALDRICH	Cat #: P8139
CP-456773 sodium salt, (MCC950)	SIGMA-ALDRICH	Cat #: PZ0280
Nigericin sodium salt	SIGMA-ALDRICH	Cat #: N7143-5MG
Monosodium Urate (MSU) Crystals	InvivoGen	Cat #: tlrl-msu
Cholesterol	SIGMA-ALDRICH	Cat #: C3045
Adenosine triphosphate, ATP	InvivoGen	Cat #: tlrl-atpl
Dextran sulfate sodium salt, 40,000	SIGMA-ALDRICH	Cat #: 42867
Suberic acid bis(N-hydroxysuccinimide ester)	SIGMA-ALDRICH	Cat #: S1885
Z-VAD FMK (CAS 187389-52-2)	Santa Cruz Biotechnology	Cat #: sc-3067
Z-YVAD-AFC	Enzo Life Sciences	Cat #: ALX-260-035-M005
Ac-YVAD-cmk	InvivoGen	Cat #: inh-yvad
Power SYBR Green PCR Master Mix	Invitrogen	Cat#: A25742
Mouse M-CSF Recombinant	Invitrogen	Cat #: PMC2044
Histopaque-1077	SIGMA-ALDRICH	Cat #: 10771 SIGMA
Trizol	Invitrogen	Cat #: 15596018
Protease Inhibitors	Roche	Cat #: 11836170001
Z-Leu-Leu-Leu-al (MG132)	SIGMA-ALDRICH	Cat #: C2211-5MG
Bafilomycin A1	InvivoGen	Cat #: tlrl-baf1
Bafilomycin A1	CST	Cat # 54645S
Earle’s Balanced Salt Solution, EBSS	Invitrogen	Cat #: 14155063
3-Metyladenine	SIGMA-ALDRICH	Cat #: M9281
Hydroxychloroquine sulfate	SIGMA-ALDRICH	Cat #: H0915-5MG
PMSF	SIGMA-ALDRICH	Cat #:P7626
2X Laemmli Sample Buffer	Bio-RAD	Cat#: 1610737

**Critical Commercial Assays**		

IL-1 beta Mouse ELISA Kit	Invitrogen	Cat #: KMC0011
IL-6 Human ELISA Kit	Invitrogen	Cat #: KHC0061
Human IL-1 beta ELISA Ready-SET GO (2^nd^ Generation)	Invitrogen	Cat #: 88-7261-88
Human TNF alpha ELISA Ready-SET GO (2^nd^ Generation)	Invitrogen	Cat #: 88-7346-88
Annexin V FITC/PI	Invitrogen	Cat #: 88-8005-74

**Deposited Data**		

Raw data of images	This paper, Mendeley Data	https://doi.org/10.17632/xx73b2kx2x.1

**Experimental Models: Cell Lines**		

THP-1	ATCC	ATCC#: TIB-202
HT-29	ATCC	ATCC#: HTB-38
HEK293T	ATCC	ATCC#: CRL-11268
THP-1 GFP ASC	InvivoGen	Cat#: thp-ascgfp

**Experimental Models: Organisms/Strains**		

C57 BL/6 irgm1^+/+^ and irgm1^−/−^ mice	Gregory A Taylor (Henry et al., 2007)	NA

**Oligonucleotides**		

IL1Beta TaqMan Gene Expression Assay (HS01555410_m1)	Thermo Fisher Scientific	Cat #: 4331182
IRGM-TaqMan Gene Expression Assay (HS01013699_s1)	Thermo Fisher Scientific	Cat #: 4331182
Human IL-18 Forward primer 5′- CCTCTATTTGAAGATATG ACTGATTCTG-3′	This manuscript	N/A
Human IL-18 Reverse primer 5′- CCATACCTCTAGGCTGGC-3′	This manuscript	N/A
Human TNF alpha Forward primer 5′-ACTTTGGAGTGATC GGCC-3′	This manuscript	N/A
Human TNF alpha Reverse primer 5′- AACATGGGCTACAG GCTTG-3′	This manuscript	N/A
Human RANTES Forward primer 5′-CTCATTGCTACTGCCC TCTG-3′	This manuscript	N/A
Human RANTES Reverse primer 5′-AATACTCCTTGATGTG GGCAC-3′	This manuscript	N/A
Mouse IL-1 beta Forward primer 5′-ACGGACCCCAAAAGA TGAAG-3′	This manuscript	N/A
Mouse IL-1 beta Reverse primer 5′-TTCTCCACAGCCACAA TGAG-3′	This manuscript	N/A
Mouse IL-18 Forward primer 5′CCTGTGTTCGAGGATATG ACTG 3′	This manuscript	N/A
Mouse IL-18 Reverse primer 5′-AGTCCTCTTACTTCACTGT CTTTG-3′	This manuscript	N/A
Mouse TNF alpha Forward primer 5′-GGAACTGGCAGAAGA GGCACTC-3′	This manuscript	N/A
Mouse TNF alpha Reverse primer 5′- GCAGGAATGAGAAGA GGCTGAGAC-3′	This manuscript	N/A
SMARTpool: siGENOME IRGM (345611) siRNA	Dharmacon	Cat #: M-028450-01-0005
SMARTpool: siGENOME ATG5 (9474) siRNA	Dharmacon	Cat #: M-004374-04-0005
Human p62 siRNA	SIGMA-ALDRICH	SASI_Hs01_00118616
Human ATG7	SIGMA-ALDRICH	SASI_Mm01_00044616
Human NLRP3 siRNA	SIGMA-ALDRICH	SASI_Hs02_00313820
Mouse NLRP3 siRNA	SIGMA-ALDRICH	SASI_Mm01_00136572

**Recombinant DNA**		

GFP-IRGM	Chauhan et. al. 2015, Vojo Deretic	N/A
Flag-IRGM	This manuscript	N/A
mCherry-IRGM	This manuscript	N/A
Flag-NLRP3 and constructs	This manuscript	N/A
GFP-NLRP3	Addgene	Plasmid # 73955
Myc-ASC	Addgene	Plasmid # 73952
HA-ASC	Addgene	Plasmid # 41553
Flag-ASC and constructs	This manuscript	N/A
pcDNA3-EGFP	Addgene	Plasmid # 13031
HA-p62	Addgene	Plasmid # 28027
Flag-p62	This Manuscript	N/A
Flag-ATG16L	Chauhan et. al. 2015, Vojo Deretic	N/A
mcherry-NACHT-NLRP3	This manuscript	N/A
GFP-AIM2	Addgene	Plasmid # 51542
HA-K48	Addgene	Plasmid # 17605
HA-K63	Addgene	Plasmid # 17606
pCMV-pro-IL-1 beta	Addgene	Plasmid # 73953
pCMV-Caspase 1-Flag	Addgene	Plasmid # 21142
pCI-Caspase 1	Addgene	Plasmid # 41552
pDest-mCherry	Terje Johansen	N/A
pDest-3X Flag	Terje Johansen	N/A

**Software and Algorithms**		

Cell Quest Pro	BD Biosciences	N/A
ImageJ	NIH	https://imagej.nih.gov/
Leica LAS AF	Leica Microsystem	N/A

**Other**		

NP-40 Lysis buffer	ThermoFisher	Cat #: FNN0021
Dynabeads Protein G	ThermoFisher	Cat #: 10004D
Protease Inhibitors Cocktail Tablets	Roche	Cat #: 11836170001
Phosstop	Roche	Cat #: 4906845001

#### Cell Culture

All the cell lines were obtained from American Type Culture Collection (ATCC). HT-29 and HEK293T cells were grown in DMEM medium (GIBCO) supplemented with 10% Fetal bovine serum (FBS). Human monocytic cell line, THP-1 and PBMC’s were grown in RPMI-1640 (GIBCO) media supplemented with 10% FBS, 5 mM L-glutamine, glucose (5%), HEPES buffer, Sodium pyruvate (1 mM), penicillin/streptomycin (10,000 units/mL). All experiments were performed with cells before the 20th passage was reached. THP1 ASC-GFP cells stably expressing an ASC::GFP fusion protein were purchased from invivogen (cat no. thp-ascgfp). These cells are derived from THP-1 human monocytic cells and the expression of ASC::GFP is driven by an NF-kB-inducible promoter that can be induced by LPS. In presence of LPS a diffused signal of ASC::GFP can be seen and only in presence of Nigericin, ASC::GFP specks were observed.

#### Isolation of human peripheral blood mononuclear cells (PBMCs)

Isolation of Human PBMCs were performed using density gradient centrifugation method using Histopaque as per manufacture recommendations (Sigma). Briefly, 5 mL homogenized blood were mixed with an equal volume of PBS and slowly layered on the 3 mL Histopaque-1077 (Sigma 1077, density 1.077). The sample was then centrifuged at 400 g for 20 minutes at room temperature (RT) using slow acceleration and deceleration. Buffy coat generated in the middle of the gradient was collected, mixed with DPBS and spun at 100 g for 10 minutes. Cells were then washed twice with DPBS and re-suspended in RPMI-1640 supplemented with 10% FBS and incubated at 37°C with 5% CO_2_ overnight. Cells were counted in an Invitrogen Countless II Automated Cell Counter using an equal volume of trypan blue (Sigma). A total of 2 × 10^6^ cells were seeded in each 6 well plate for treatment with LPS (100 ng/ml). Total RNA was extracted using TRIZOL (Invitrogen).

#### Plasmid Constructs and Transfection

The mcherry-IRGM and Flag-NLRP3 and its deletion constructs were generated using gateway cloning strategy as per standard protocols (Invitrogen).

#### Transient transfection with siRNA

The THP-1 and PBMC cells were electroporated (Neon, Invitrogen; setting: 1400V, 10ms, 3 pulse using the 100 μl tip) with non-targeting siRNA (30nM) or IRGM siRNA (30nM) or p62 siRNA (30nM) or ATG5 siRNA ((30nM) or ATG7 siRNA ((30nM) and incubated for 24h. After 24h, one more time transfection was performed in similar condition as described above and incubated for another 48 h before start of each experiment. The HT-29 cells and HEK293T cells were transfected with 10 nM siRNA using Lipofectamine® RNAiMAX Transfection Reagent as per manufacturer’s instructions.

For transient gene/s knockdown experiment in BMDMs: Freshly isolated, 4x10^6^ Bone marrow cells electroporated with non-targeting siRNA or NLRP3 siRNA (30nM) using Neon system (Invitrogen) with following parameter: 1350V, 10 ms. 3 pulse and kept for differentiation with 20ng/ml Mouse M-CSF in media into macrophages for 4 days. After 4 days, BMDMs cells were again transfected with non-targeting siRNA or NLRP3 siRNA (30nM) using Lipofectamine® RNAiMAX transfection Reagent as per manufacturer’s instructions.

#### Transient transfection with plasmids

HEK293T cells for overexpression experiment were transfected using calcium phosphate method as per the manufacturer’s instructions (Clonetech, Promega).

Transfection in THP-1 or THP-1 GFP ASC cells: After 72 h of ATG5 (30nM) or ATG7 (30nM) or p62(30nM) siRNA transfection, 2x10^6^ THP-1 cells were transfected with EGFP (3 μg) and GFP-IRGM (2 μg) using the Neon electroporation system (Invitrogen) with following parameter: 1300 V, 30 ms, 1 pulse. After 1 h of post-transfection cells were stimulated with LPS (1 μg) and incubated for 3h. Cell lysates were prepared and western blots were performed.

Inflammasome reconstitution: 3x10^5^ HEK293T cells were plated in 6 well plate and transfected with NLRP3, HA-ASC, Caspase-1, pro-IL1 beta, GFP or GFP IRGM plasmids for 30 h. After 30 h cells were replenished with fresh medium without serum with or without Chloroquine (50 μM) and incubated for another 6h. After incubation, supernatant was collected and used for IL-1β release and cells lysate was used for immunoblotting with different antibodies.

#### Enzyme-linked immunosorbent assay (ELISA)

ELISA was performed using the Affymetrix (ebiosciences) Kit as per manufacturer’s instructions. Briefly, polystyrene 96-well plates (Corning Costar # 9018) were pre-coated overnight at 4°C with specific capture antibody, then blocked with Elispot blocking (diluent) buffer for 1 h at RT, and incubated with standard cytokine dilutions and cell cultures media (100 μl, undiluted) for O/N at 4°C followed by washes with PBS plus 0.05% Tween 20, and incubation with biotinylated detection antibody for 1h at RT. After the second wash, the plates were incubated with HRP-Avidin for 30 min at RT and washed again. The signal was developed after addition 1X TMB (3,3′,5,5′-Tetramethylbenzidine) until color appeared and the reaction was stopped by 1M H_3_PO_4_. Microplate reader (Bio-RAD) was used to detect the signals at 450 nm.

#### Bacterial Infections

A single colony of *Salmonella typhimurium* strain *S*T 1433 strain was grown in antibiotic-free Luria Bertani (LB) media until OD_450_ reached 0.5- 0.6. Prior to infection, bacterial cells were washed thrice with 1 mL of sterile PBS and re-suspended in antibiotic-free DMEM or RPMI medium. THP-1, and HT29 cells were infected at multiplicity of infection (MOI) of 10:1 (bacteria: cells).

#### ASC Oligomerization Assay

The THP-1 control and IRGM depleted cells or BMDM cells were seeded in 6 well plates and stimulated with LPS (1 μg/ml) for 3 h followed by nigericin (10 μM) for 30 min. Cells were lysed with buffer containing 0.5% Triton X-100, and the cell lysates were centrifuged at 6,797 g for 15 min at 4°C. Supernatants were transferred to new tubes (Triton-X-soluble fractions). The Triton X-100-insoluble pellets were washed with PBS twice and then suspended in 200 μL PBS. The pellets were then cross-linked at room temperature for 30 min by adding 2 mM suberic acid bis (N-hydroxysuccinimide ester), (DSS). The cross-linked pellets were spun down at 6,797 g for 15 min and dissolved directly in non- reducing SDS sample buffer.

#### Western Blotting

The NP-40 (Invitrogen) or Radio-immunoprecipitation assay (RIPA) buffer (20 mM Tris, pH 8.0; 1mM, EDTA; 0.5 mM, EGTA; 0.1% Sodium deoxycholate; 150 mM NaCl; 1% IGEPAL (Sigma);10% glycerol) supplemented with protease inhibitor cocktail (Roche) and 1mM PMSF(Sigma) was used to make cell lysates. Protein concentration was measured by BCA kit (Pierce). Protein lysates were separated on SDS-polyacrylamide gel, transferred onto nitrocellulose membrane (Bio-Rad) and blocked for 1 h in 5% skimmed milk. Subsequently, membranes were incubated in primary antibody overnight at 4°C, washed (3 X PBS/PBST) and then incubated for 1 h with HRP conjugated secondary antibody. After washing with PBS/PBST (3X) the blots are developed using enhanced chemiluminescence system (Thermo Fisher). Densitometric analysis of western blots were done using ImageJ software.

#### Antibodies and dilution

##### Primary antibodies used in western blotting with dilutions

Flag (1:1000), c-Myc (Santacruz-SC-40; 1:750 and sc-764; 1:1000), p62 (BD; 1:2000), Actin (Abcam; 1:5000), IRGM (Abcam; 1:500), HA (CST; 1:1000), IL-1β (CST; 1:1000), Caspase-1(Adipogen; 1:1000), NLRP3 (Adipogen; 1:1000), GFP (Abcam; 1:5000), ASC (Santacruz; 1:1000), ATG-5 (CST, 1:1000), ATG7 (CST, 1:1000), NLRC4 (CST; 1:1000) and AIM2 (CST; 1:1000), IRGM Antibody Rodent specific (CST; 1:1000), c-Myc (Santa Cruz; 1:750). Phsopho-p38 MAPK (CST, 1:1000), p-38α (Santa cruz, 1:1000), Anti-pro Caspase1+p10+p12 Antibody (Abcam; 1:1000), NF-kb p65 (Santa cruz; 1:1000), p- NF-kb p65 (Santa cruz; 1:1000).

HRP conjugated secondary antibodies were purchased from Santa cruz (1:2000) or Promega (1:5000) or Abcam (1:10000).

##### Primary antibodies used in immunofluorescences with dilutions

Flag (Sigma; 1:250), IRGM (Abcam; 1:100), NLRP3 (Adipogen; 1:200), NLRP3 (CST; 1:400), c-Myc (Santa Cruz;1:50), p62 (BD; 1:500); ASC (Adipogen; 1:200), HA (CST; 1:500).

#### Co-Immunoprecipitation

For immunoprecipitation assays cells were lysed in NP-40 lysis buffer (FNN0021, Thermo Fisher Scientific) supplemented with protease inhibitor/phosphatase inhibitor cocktails and 1M PMSF for 20 min at 4°C and centrifuged. The supernatant was incubated with antibody at 4°C (2 h to O/N) on rotational cell mixer followed by incubation with Protein G Dynabeads (Invitrogen, #10004D) for 2 h at 4°C. The beads were washed with ice cold PBS (4X) and the proteins were eluted from washed beads by boiling for 5 min in 2X SDS-PAGE gel loading dyeand proceeded for western blot analysis.

#### GST pull-down assay

GST pulldown assay was done as described previously (Kimura et al., 2015, Chauhan et al., 2016). GST-IRGM recombinant protein was expressed in SoluBL21 (Amsbio) and purified on Glutathione Sepharose 4 Fast-Flow beads (GE Healthcare). [35S]-labeled Myc-NLRP3 was cotranscribed/translated using the TnT T7–coupled reticulocyte lysate system (Promega). The *in vitro*–translated [35S]-labeled Myc-NLRP3 protein was then incubated with GST or GST-IRGM in 250 μl of NETN-E buffer (50 mm Tris, pH 8.0, 100 mm NaCl, 6 mm EDTA, 6 mm EGTA, 0.5% NP-40, and 1 mm dithiothreitol supplemented with complete mini EDTA-free protease inhibitor cocktail [Roche]) for 2 h at 4°C and then washed five times with 1 mL of NETN-E buffer, boiled with loading buffer, and subjected to SDS-PAGE. Gel was stained with Coomassie Blue and vacuum-dried. The GST-IRGM was detected by staining with Coomassie Blue, whereas the [35S]-labeled Myc-NLRP3 was detected in PharosFX imager (Bio-Rad Laboratories)

#### Immunofluorescence Analysis

About 10^5^ cells were plated on a coverslip. The next day, cells were fixed in 4% paraformaldehyde for 10 minutes (min), permeabilized with 0.1% saponin (or 0.1% Triton X-100) for 10 min, followed by blocking with 1% BSA for 30 min at room temperature (RT). Permeabilized cells were then incubated with primary antibody for 1 hour (h) at RT, washed thrice with 1X PBS, followed by 1 h incubation with Alexa Flour conjugated secondary antibody. Cells were again washed thrice with 1X PBS, mounted (Prolong gold antifade, Invitrogen), air-dried, and visualized using Leica TCS SP5 confocal microscope.

#### Cycloheximide chase assay

After the plasmids transfection, cells were treated with MG132 (20 μM) or Bafilomycin (300 nM) along with cycloheximide (100 μg/ml) for 2, 4, 6 and 8 h. At various time points cells were lysed in NP-40 lysis buffer and western blots were performed with indicated antibodies.

#### Macrophage differentiation and stimulation

For immunofluorescence assays, THP-1 cells were differentiated into macrophage-like state by addition of 50 ng/ml of PMA (16 h). After a resting period of 48 h macrophages were treated with stimulants as required.

#### Chloroform-methanol protein precipitation

Proteins from cultured media supernatants were isolated by methanol/chloroform precipitation assay. Briefly, 500 μL of supernatant was mixed with 500 μL of methanol and 125ul of chloroform and mixed. After centrifugation, the aqueous phase was removed without disturbing the protein layer. An additional 500 μL of methanol was added to the sample and centrifuged for protein precipitation. The protein pellet was air-dried and re-suspended in of 2x Laemmli buffer. The sample was boiled at 95°C for 5min and separated on a 12% SDS-polyacrylamide gel and transferred to the nitrocellulose membrane.

#### Annexin-V/Propidium Iodide staining

Apoptosis is measured using Annexin-V/PI double staining method as per manufacturer’s instruction (ebiosciences #88800574). Briefly, cells were treated with required stress-inducers and followed by desired incubation time cells were washed with PBS and trypsinized to obtain a single cell suspension. Next, 10^5^ cells/ml were suspended in 100 μL binding buffer along with 5 μL Annexin V-fluorescein isothiocyanate and 5 μL Propidium Iodide for 30 min at room temperature followed by acquisition on FACS Calibur (Beckton & Dickinson). The results were analyzed using Cell Quest Pro software.

#### Caspase-1 inhibition Assay

To inhibits Caspase 1 activity, THP-1 or BMDM cells were treated with Caspase Inhibitor (Z-VAD FMK, 5 μM) or caspase 1 specific inhibitor (Ac-YVAD-cmk, 20 μM) 30 min prior to stimulation as indicated. Cells were processed for apoptosis assay through Annexin V/PI staining as described above or western blots for various proteins.

#### Soluble and Insoluble proteins fractionation

Soluble-insoluble proteins fractionation was performed using buffer containing 2% Triton X-100, 50 mM Tris (pH 8.0), 150 mM NaCl, 1mM EDTA, 10% glycerol, protease inhibitor cocktail and 1mM PMSF. After centrifugation, the supernatant is used as soluble fraction and the pellet was extracted in 1% SDS to obtain insoluble protein fraction.

#### RNA Isolation and Quantitative Real-time PCR

The total RNA was extracted using Trizol reagent according to manufacturer’s protocols (Invitrogen). 2 μg of RNA was used for reverse transcription using high capacity DNA reverse transcription kit (Applied Biosystems Cat No: 4368813) and qRT-PCR was performed using Taqman master mix or Power SYBR green PCR master mix (Applied biosystems cat No: 4367659) according to manufacturer’s protocols on Step One Real-Time PCR system (Applied Biosystems). The following cycling program was used for SYBR green assay: initial denaturation at 95°C for 10 min; 40 cycles of 95°C for 15 s and 60°C for 60 s; followed by a melt curve step. For Taqman assay: Initial denaturation 50°C for 2 min and 95°C for 10 min; 40 cycles of 95°C for 15 s and 60°C for 60 s. mRNA expression profiles were normalized to levels of housekeeping gene Glyceraldehyde 3-phosphate dehydrogenase (GAPDH) or β-Actin in each sample and the fold-change in expression was calculated by the 2^−ΔΔCt^ method.

#### Caspase 1 activity measurement assay

For caspase-1 activity assay, BMDM’s or THP-1 cells were primed with LPS and treated with Nigericin and colon tissue lysed in lysis buffer (0.1% Nonidet P-40, 1mM EDTA, 50mM HEPES, pH7.5, 0.1 CHAPS,1mM PMSF, 2mM dithiothreitol and protease Inhibitors cocktail). Equal amount (20 μg from each sample) of protein transferred to each well of a 96-well microplate in triplicate. The volume in each well-adjusted with 50μl/well with cell lysis buffer. 50 μl lysis buffer were taken for background subtraction. 50 μl caspase 1 buffer (100 mM HEPES, 10% Sucrose, 0.1% CHAPS, 1mM Na-EDTA and 2mM dithiothreitol) with Z-YVAD-AFC substrate (50 μM final concentration) added to each well and incubated at 37°C in dark. Free AFC was measured on a fluorescence microplate reader at Ex/Em = 400/505.

#### Mice experiments

*Irgm1* knock out (C57BL/6) mice were described previously (Liu et al., 2013). The mice experiments were performed with procedures approved by institutional animal ethical committee at Institute of Life Science, Bhubaneswar, India. For each experiment, littermates were used and also gender and age of mice were matched. For Dextran Sulfate Sodium (DSS) induced colitis model, the mice were given with 2% (wt/vol) of DSS dissolved in drinking water. DSS solution was replaced on alternate day. To inhibits the NLRP3, MCC950 (20 mg/Kg) injected intraperitoneally (IP) into the mice. The control group were injected intraperitoneally with saline. Each day, mice were monitored for body weight and stool consistency. In brief, stool scores were assigned as follows: 0, well-formed pellets; 1, semi solid; 2, semi solid and not adhere to the anus; 3, liquid and adhere to the anus; 4, diarrhea. Bleeding scores were assigned as follows: 0, No blood in stool; 1, light faint; 2, clear visible; 3 gross rectal bleeding. The mice were sacrificed and entire colon was removed and colon length was measured using Vernier scale. Approx. 0.5 cm colon tissue near to rectum was collected and homogenized in RIPA buffer containing protease inhibitors cocktails for protein extraction and western blotting or homogenized in trizol for total RNA extraction.

#### Mice Bone marrow cells isolation and differentiation into macrophages

The bone marrow cells from mice were isolated and differentiated into macrophages by standard procedure (Henry et al., 2007). Briefly, six to eight week old male C57 BL/6 irgm1^+/+^ and irgm1^−/−^ mice were sacrificed by cervical dislocation, bone marrow cells from the tibia and femurs were flushed out in RPMI medium. Red blood cells were removed by cell lysis buffer containing (155 mM NH_4_Cl, 12 mM NaHCO_3_ and 0.1 mM EDTA). Cells were differentiated in RPMI medium (10% FBS, 1mM sodium pyruvate and 0.05 M 2-mercaptoethanol) with 20 ng/ml Mouse M-CSF (GIBCO PMC2044, ThermoFisher Scientific) for 5 days. On alternate day, medium was replaced with fresh medium containing M-CSF.

### Data and Software Accessibility

All the original data for western blotting and Immunofluorescence have been deposited with Mendeley and can be accessed with https://doi.org/10.17632/xx73b2kx2x.1.

## Supplemental Information

Document S1. Figures S1–S7

Document S2. Article plus Supplemental Information
