# Supplementary material for: The Crohn’s Disease Risk Factor IRGM Limits NLRP3 Inflammasome Activation by Impeding Its Assembly and by Mediating Its Selective Autophagy
Source: Mol Cell. 2019 Feb 7;73(3):429–445.e7. doi: 10.1016/j.molcel.2018.11.018 (PMC6372082; doi:10.1016/j.molcel.2018.11.018)
Supplement: Document S1. Figures S1–S7 [file mmc1.pdf]

Molecular Cell, Volume *73*

Supplemental Information

The Crohn's Disease Risk Factor IRGM Limits NLRP3 Inflammasome Activation by Impeding Its Assembly and by Mediating Its Selective Autophagy

Subhash Mehto, Kautilya Kumar Jena, Parej Nath, Swati Chauhan, Srinivasa Prasad Kolapalli, Saroj Kumar Das, Pradyumna Kumar Sahoo, Ashish Jain, Gregory A. Taylor, and Santosh Chauhan

Molecular Cell Volume

Supplemental Information

The Crohn’s disease risk factor IRGM limits NLRP3 inflammasome activation by impeding its assembly and by mediating its selective autophagy

Subhash Mehto, Kautilya Kumar Jena, Parej Nath, Swati Chauhan, Srinivasa Prasad Kolapalli, Saroj Kumar Das, Pradyumna Kumar Sahoo, Ashish Jain, Gregory A Taylor, and Santosh Chauhan

Supplementary Figure 1

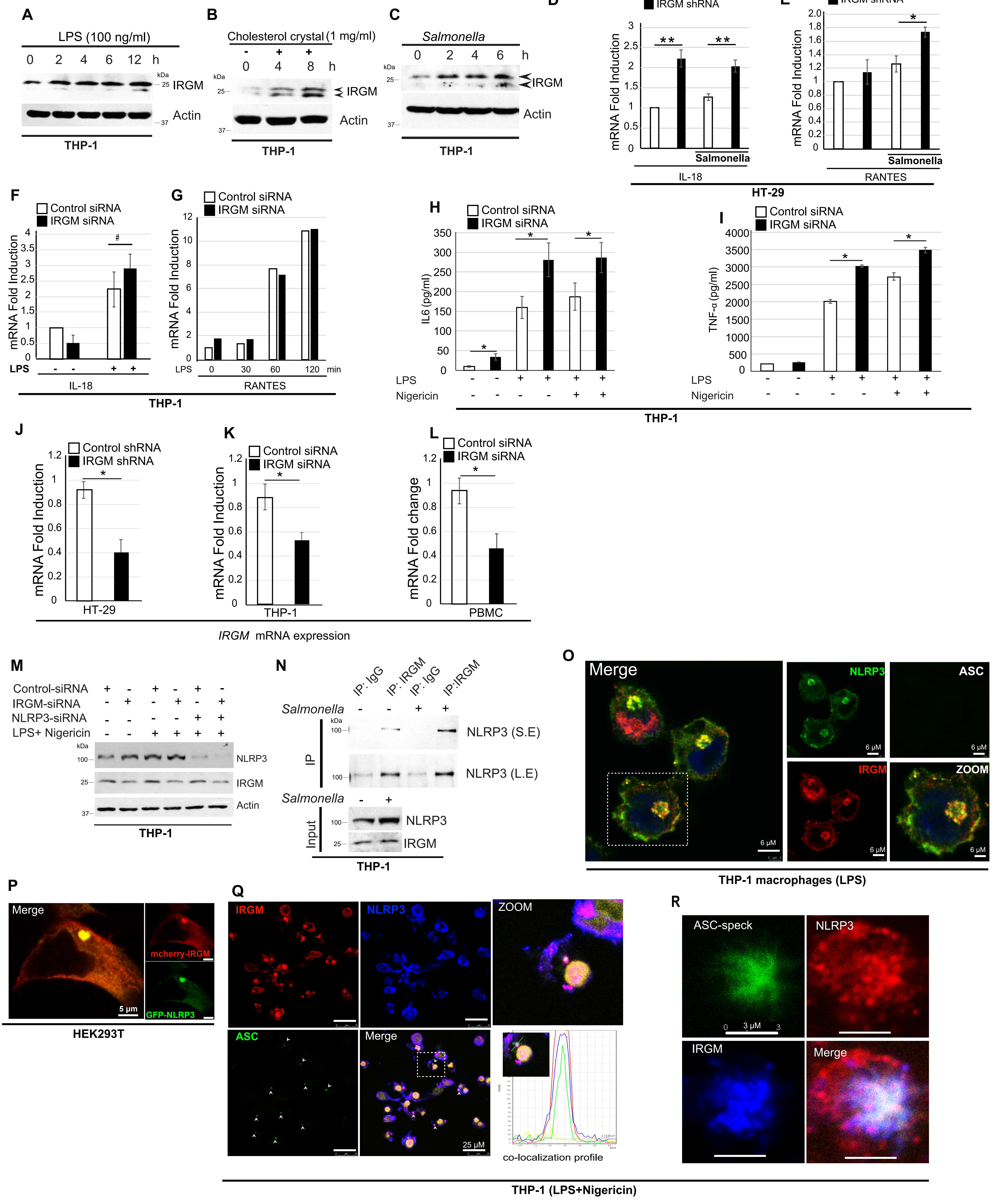

Figure S1. IRGM inhibits the pro-inflammatory response and the activation NLRP3-inflammasome, Related to Figure 1 and Figure 2. (A, B) THP-1 cells were stimulated with LPS (100 ng/ml) (A) and inflammasome inducers (B) cholesterol crystals (1 mg/ml) for indicated time periods and protein extracts were subjected to western blotting with IRGM antibody. (C) THP-1 cells were infected with *Salmonella typhimurium* (SL1433) at a MOI of 1:10 for indicated time periods and protein extracts were subjected to immunoblotting with indicated antibodies. (D, E) HT-29 control and IRGM knockdown cells were infected with *S. typhimurium* (1:10 MOI, 8 h) and the total RNA was isolated and subjected to qRT-PCR for indicated genes. (n=3, Mean ±SD, $*p < 0.05$, $**p < 0.005$, Student's unpaired t test). (F, G) The total RNA isolated from the LPS stimulated (100 ng/ml, 2 h) control and IRGM siRNA transfected THP-1 cells was subjected to qRT-PCR for the indicated genes.$^{\#}$insignificant. (H, I) The supernatants from control and IRGM siRNA transfected THP-1 cells which were stimulated with LPS (100 ng/ml, 4 h) alone or in combination with nigericin (5 μM, 30 min) were subjected to ELISA with IL-6 (H) and TNF-α antibody (I). (n=3, Mean ±SD, $*p < 0.05$). (J, K, L) IRGM knockdown efficiency was analysed using qRT-PCR from control and IRGM knockdown HT-29 cells (J), THP-1 cells (K), and Human PBMCs (L). (for J, K panels, n=3, Mean ±SD, $*p < 0.05$; for L panel, n=5, Mean ±SE, $*p < 0.05$). (M) Knockdown efficiency check of IRGM and NLRP3 for panel O and P of Figure 1. (N) IP analysis of interaction between endogenous IRGM and NLRP3 in uninfected or S. typhimurium infected (2h) THP-1 cells. (O) Representative confocal images of THP-1 macrophages, treated with LPS (100 ng/ml, 4h) and processed for immunofluorescence (IF) analysis with NLRP3, IRGM and ASC antibodies. (P) Representative confocal images of HEK293T cells transiently expressing mcherry-IRGM and GFP NLRP3. (Q, R) Representative confocal images of THP-1 cells, treated with LPS (1 μg/ml, 3h) and nigericin (5 μM, 15 min) and processed for immunofluorescence (IF) analysis with NLRP3, ASC and IRGM antibodies. Scale bar are indicated in the respective figures.In all panels, S.E, Short exposure; L.E, Long exposure.

Supplementary Figure 2

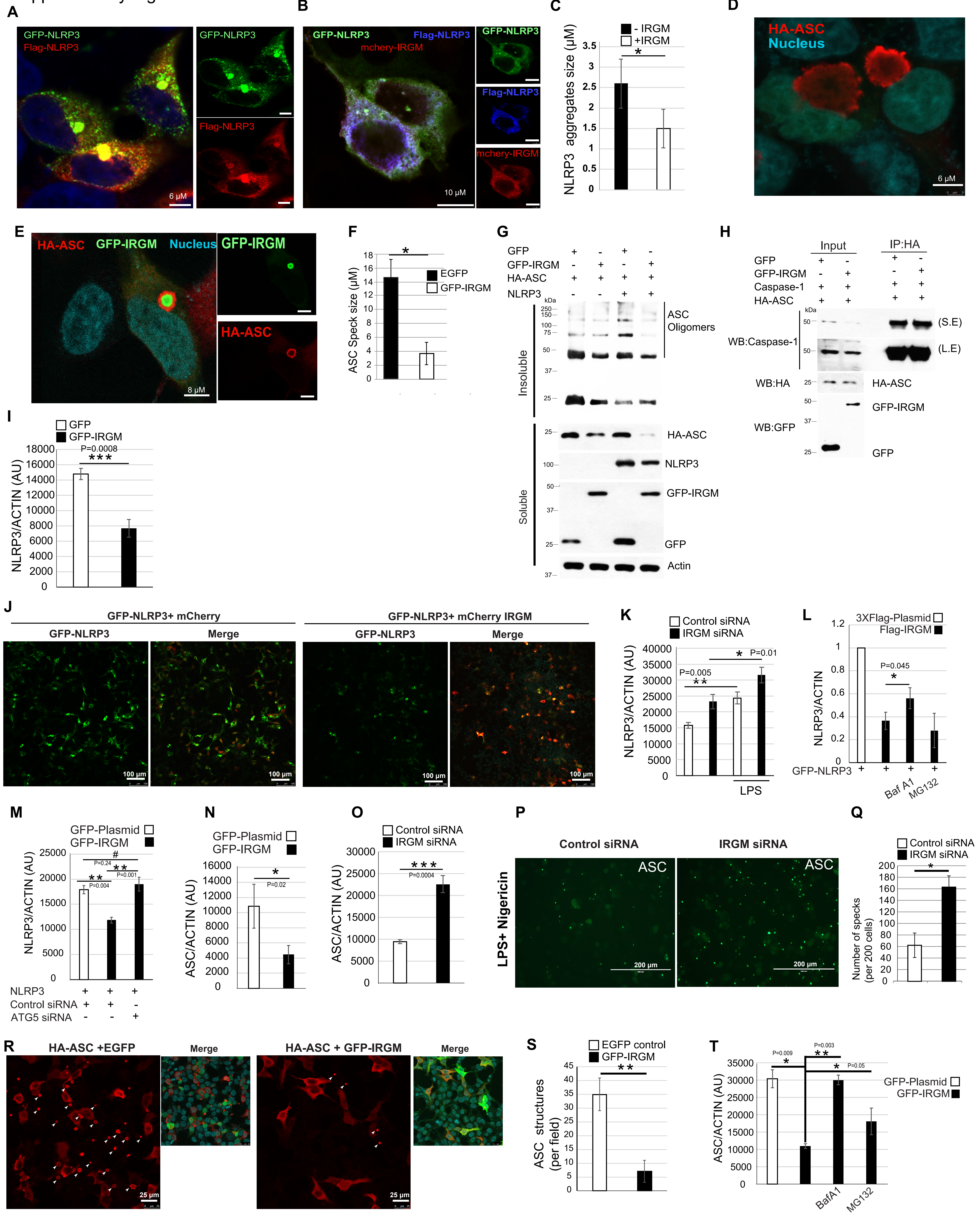

Figure S2. IRGM impedes assembly and induces autophagy of NLRP3 Inflammasome, Related to Figure 3 and Figure 4. (A, B, C) Representative confocal images of HEK 293T cells transiently expressing Flag-NLRP3 and GFP NLRP3 in absence (A) and in the presence of mcherry-IRGM (B). Graph depicts the average size of NLRP3 aggregates in absence and presence of IRGM (25 different NLRP3 aggregate size were measured from 3 different fields, n=2, Mean ±SD, *p < 0.05) (C). (D, E, F) Representative confocal images of HEK293T cells transiently expressing HA-ASC alone (D) or with GFP-IRGM (E). (F) Graph depicts the average size of ASC aggregates in absence and presence of IRGM (30 different ASC structure were measured from 3 different fields, n=2, Mean ±SD, *p < 0.05). (G) The soluble and insoluble fractions of DSS cross-linked HEK293T cells lysates expressing HA-ASC, NLRP3 and GFP or GFP-IRGM were subjected to Western blotting with indicated antibodies. (H) Co-IP assay for interaction between HA-ASC and Caspase-1 in presence of GFP or GFP-IRGM in HEK293T cells lysates. (I) Graph depicts the NLRP3 band intensity (normalized compared to actin) (related to Figure 4A) as measured using Image J software, (n=3, Mean ±SD, ***p < 0.0005, Student's unpaired t test) (J) Confocal images of HEK293T cells expressing GFP-NLRP3 in presence of mcherry or mcherry-IRGM. (K) Graph depicts the quantification of NLRP3 band intensity (normalized compared to actin) (related to Figure 4B) as measured using Image J software, (n=3, Mean ±SD, *p < 0.05 **p = 0.005, Student's unpaired t test). (L) Graph depicts the ratio of NLRP3 band intensity (normalized compared to actin) in different samples (related to Figure 4C). Intensity was measured using Image J software (n=3, Mean ±SD, *p < 0.05, Student's unpaired t test. (M) Graph depicts the quantification of NLRP3 band intensity (normalized compared to actin) (related to Figure 4D) as measured using Image J software, (n=3, Mean ±SD, **p <0.005, #Insignificant, Student's unpaired t test). (N) Graph depicts the quantification of ASC band intensity (normalized compared to actin) (related to Figure 4H) as measured using Image J software, (n=3, Mean ±SD, *p < 0.05, Student's unpaired t test). (O) Graph depicts the quantification of ASC band intensity (normalized compared to actin) (related to Figure 4I) as measured using Image J software, (n=3, Mean ±SD, ***p < 0.0005, Student's unpaired t test). (P) Representative confocal images of THP-1 cells stably expressing LPS-inducible GFP-ASC which were transfected with control siRNA and IRGM siRNA and followed by treatment with LPS (1 µg/ml, 3h) and nigericin (5 µm, 15 min). (Q) Graphs depicts the average number of specks formed per 200 cells counted from three to four 40X magnifications fields. (n=3, Mean ±SD, *p < 0.05). (R) Confocal images of HEK293T cells expressing HA-ASC in presence of EGFP or in presence of GFP-IRGM. (S) Graphs depicts the average number of HA-ASC structures per field (63X magnifications). (10 fields, n=3, Mean ±SD, **p < 0.005). (T) Graph depicts the quantification of ASC band intensity (normalized compared to actin) (related to Figure 4J) as measured using Image J software, (n=3, Mean ±SD, *p ≤ 0.05, **p < 0.005, Student's unpaired t test). All the experiments shown in this figure are representative of three independent experiments unless otherwise stated above.

Supplementary Figure 3

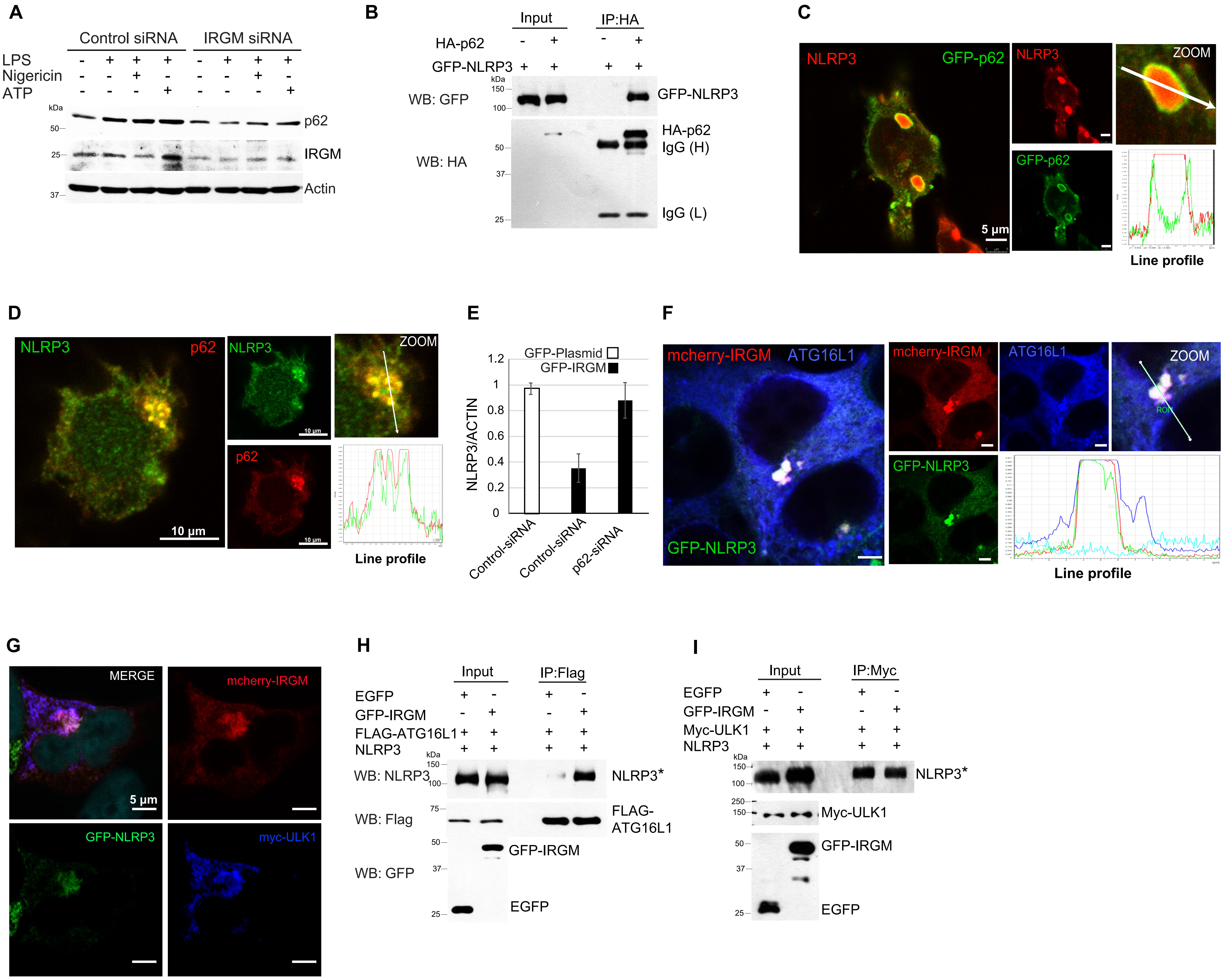

Figure S3. IRGM mediates p62-dependent selective autophagic degradation of NLRP3 and ASC, Related to Figure 4. (A) The control and IRGM siRNA transfected THP-1 cells which were stimulated with LPS (1 µg/ml, 3 h) alone or in combination with nigericin (5 µM, 30 min) or with ATP (2.5 mM) were subject to immunoblotting with indicated antibodies. (B) Co-IP analysis of interaction between p62 and NLRP3 in HEK293T cell expressing plasmids. (C) Representative confocal images of HEK293T cells expressing GFP-p62 and NLRP3. (D) Representative confocal images of THP-1 macrophages, treated with LPS (100 ng/ml, 4 h) and processed for immunofluorescence (IF) analysis with NLRP3 and p62 antibodies. (E) Graph depicts the quantification of NLRP3 band intensity (normalized compared to actin) (related to Figure 4Q) as measured using Image J software, (n=2, Mean ±SD). (F) Representative confocal images of HEK293T cells expressing mcherry-IRGM, Flag-ATG 16L1, and GFP-NLRP3. (G) Representative confocal images of HEK293T cells expressing mcherry-IRGM, myc-ULK1, and GFP-NLRP3. (H, I) Co-IP analysis of interaction between NLRP3 and ATG16 L1 (H) or NLRP3 and ULK1 (I) in absence and presence of IRGM in HEK293T cells expressing the indicated plasmids. *In order to reduce the artefact coming from IRGM-mediated degradation of NLRP3, the inputs ratios were adjusted so as to have equal inputs in both the conditions and the IP samples was run in the same ratios as of the inputs. All the experiments shown in this figure are representative of three independent experiments unless otherwise stated above.

Supplementary Figure 4

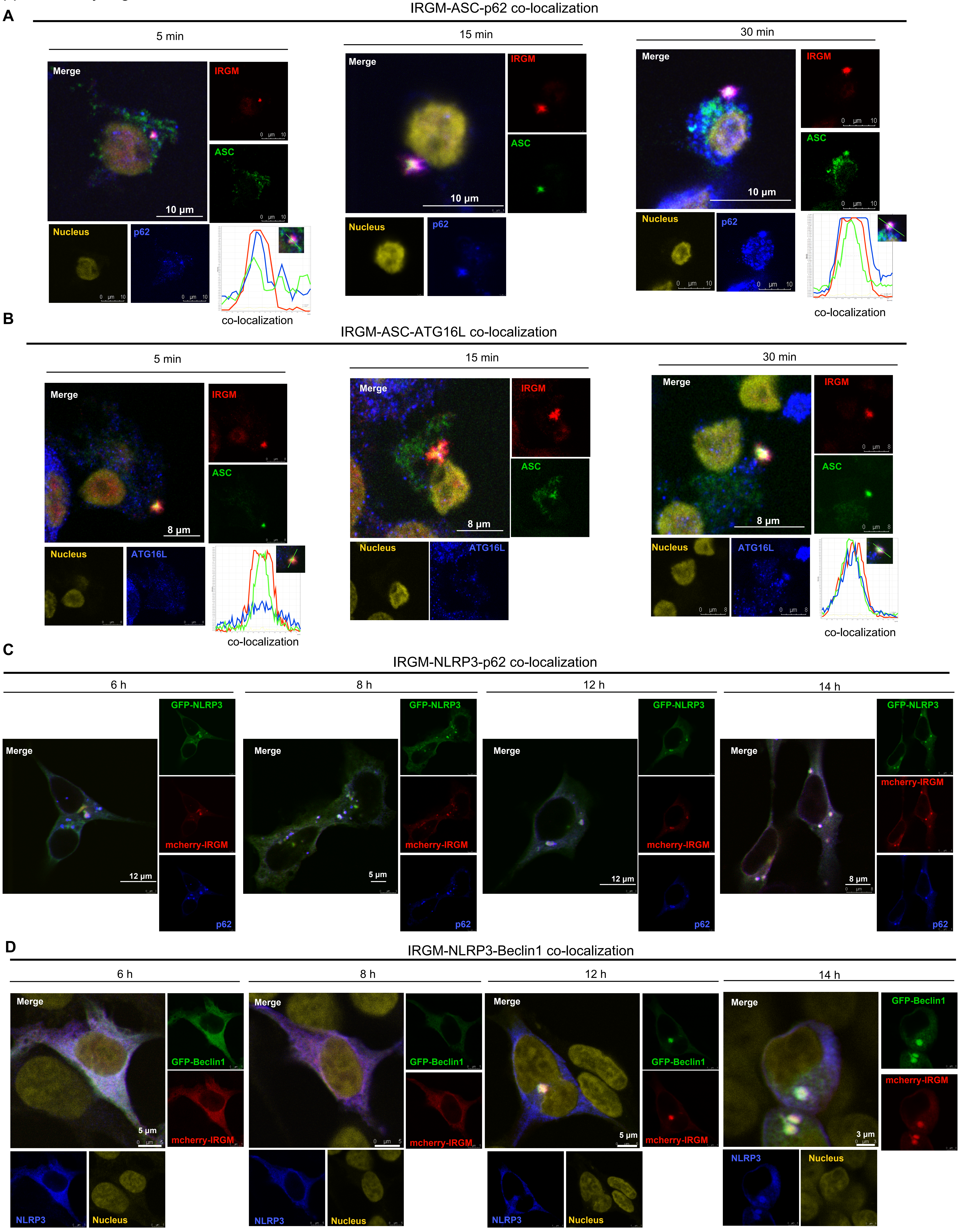

Figure S4. IRGM enhances the interaction of autophagy machinery with NLRP3 inflammasomes, Related to Figure 5. (A, B) Representative confocal images of time-chase IF experiment performed with GFP ASC stable cells stimulated with LPS (1μg/ml, 3 h) and nigericin (5 μM) and stained with ASC, IRGM and p62 (A) or ATG16L (B) antibodies. (C, D) Representative confocal images of time-chase IF experiment performed with HEK293T cells transfected with mcherry IRGM, GFP NLRP3, and HA-p62 (C) or GFP Beclin1 (D).

Supplementary Figure 5

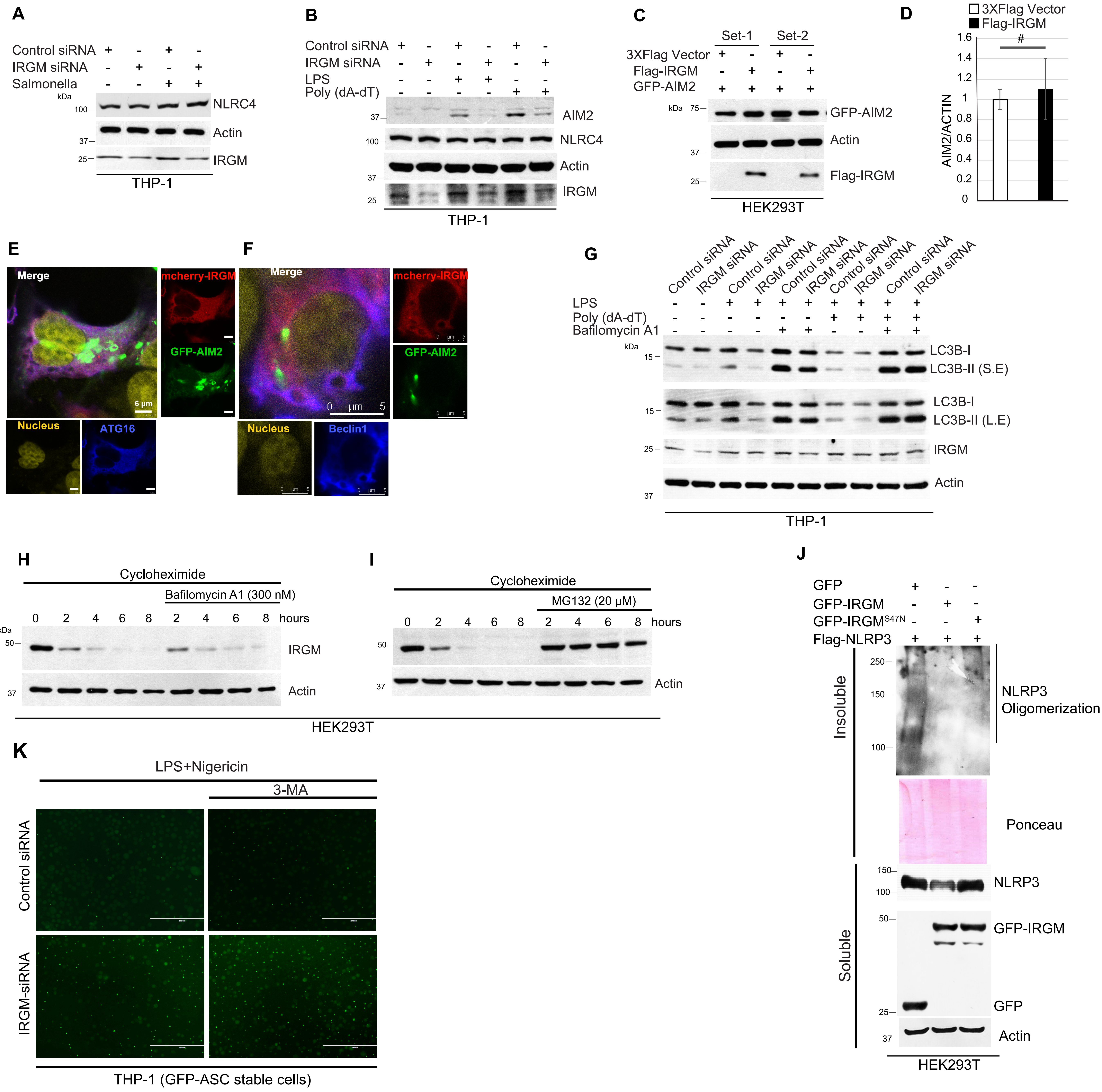

Figure S5. IRGM specifically degrades NLRP3 but not NLRC4 or AIM2, Related to Figure 4, and Figure 5. (A) The control and IRGM siRNA transfected THP-1 cells which were infected with *Salmonella typhimurium* and immunoblotting was performed with lysates using antibodies as indicated. (B) THP-1 cells transfected with control and IRGM siRNA and stimulated with LPS (1 µg/ml, 3 h and Poly(dA-dT)/Lyovec (1µg/ml, 4 h), immunoblotting was performed with lysates using antibodies as indicated. (C) HEK293T cells transfected with GFP-AIM2, Flag-IRGM and 3X Flag plasmids for 16h, immunoblotting was performed with lysates using antibodies as indicated. (D) Graph depicts the quantification of GFP-AIM2 band intensity (normalized compared to actin) as measured using Image J software, (n=3, Mean ±SD, #Insignificant). (E, F) Representative confocal images of HEK293T transfected with GFP-AIM2, mcherry-IRGM and Flag-ATG16L (E) or Flag-Beclin1 (F). (G) The control and IRGM siRNA transfected THP-1 cells stimulated with LPS (500 ng/ml, 3h), Poly (dA-dT)/Lyvovec (1 µg/ml, 4 h) and Bafilomycin A (300 nM, 4 h). S.E, Short exposure; L.E, Long exposure. (H, I) Western blot analysis GFP-IRGM transfected HEK293T cells treated with cycloheximide (100 µg/ml) and (H) 300 nM Bafilomycin A1 or (I) 20 µM Mg132 for the indicated period and blotting is performed with GFP and Actin antibodies. (J) The DSS cross-linked insoluble and soluble fraction of HEK 293T cells transfected with Flag NLRP3, GFP-IRGM and GFP-IRGM S47N were subjected to western blotting with indicated antibodies. (K) Representative images of control and IRGM siRNA transfected THP-1 GFP ASC stable cells which were stimulated with LPS (1µg/ml) and nigericin (2 µM, 30 min) and 3-MA (10 mM) as indicted.

Supplementary Figure 6

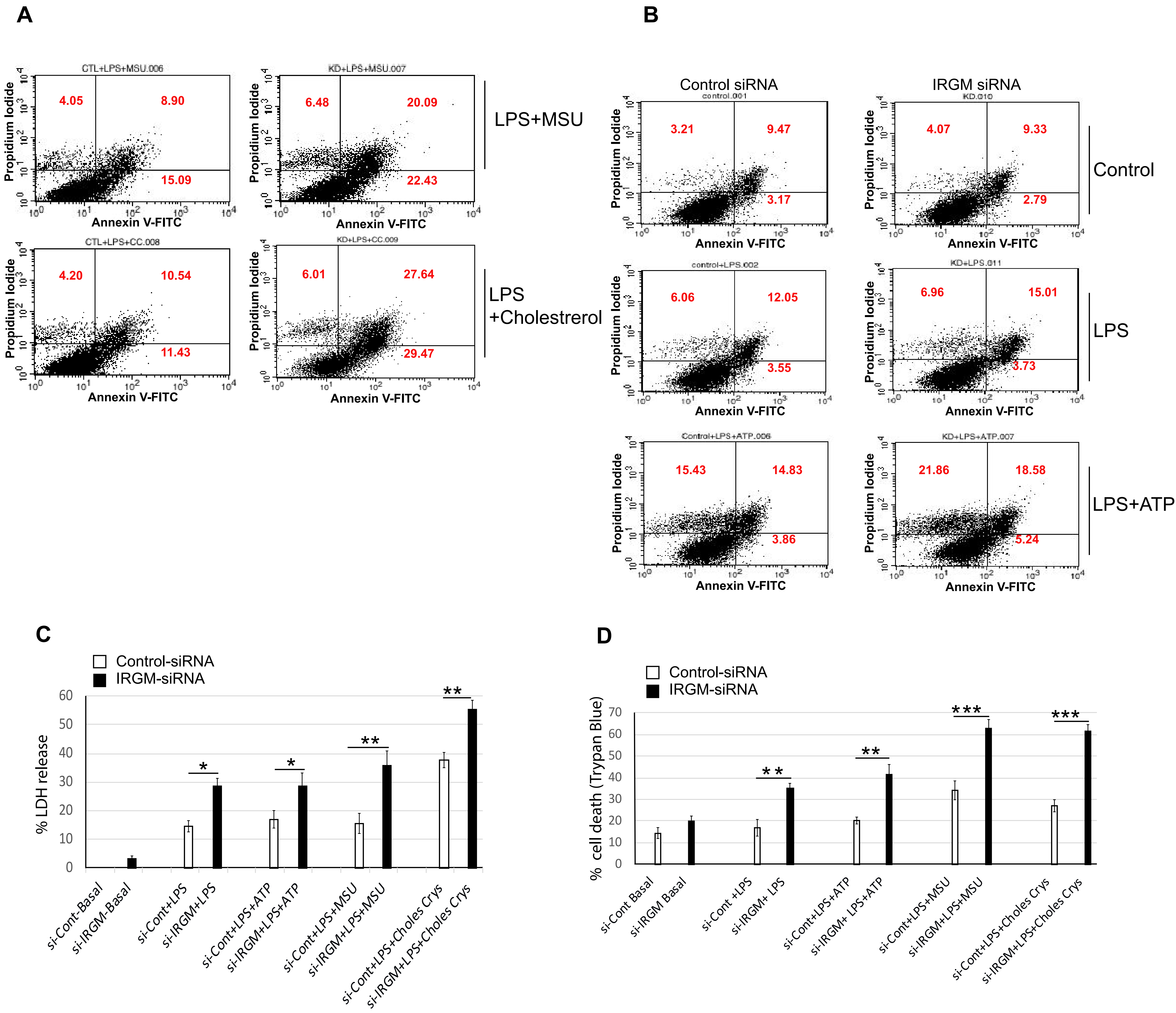

Figure S6. IRGM protects from pyroptotic cell death, Related to Figure 6. (A, B) Representative flow cytometry dot blots of control and IRGM siRNA knock down cells stained with Annexin-V/Propidium Iodide (double staining), untreated or treated with LPS (1 µg/ml, 3 h), LPS (1 µg/ml, 3 h) and MSU (200 µg/ml, 12 h) and LPS (1 µg/ml, 3 h) and Cholesterol (1 mg/ml, 12 h) (A), LPS (1 µg/ml, 3h), LPS (1 µg/ml, 3h) and ATP (5 mM, 8 h). (C) The graph depicts percentage of LDH release (compared to completely lysed samples whose readings are taken as 100% release) from the control and siRNA transfected THP-1 cells untreated or treated with LPS (1 µg/ml, 3 h), LPS (1 µg/ml, 3 h) and ATP (5 mM, 8 h), LPS (1 µg/ml, 3 h) and Cholesterol (1 mg/ml, 12 h), and LPS (1 µg/ml, 3 h) and MSU (200 µg/ml, 12 h) (n=3, Mean ±SD, *p ≤ 0.05, **p ≤ 0.005, Student's unpaired t test). (D) Trypan blue dye exclusion assays depicting percentage of cell death from the control and siRNA transfected THP-1 cells untreated or treated with LPS (1 µg/ml, 3 h), LPS (1 µg/ml, 3 h) and ATP (5 mM, 8h), LPS (1 µg/ml, 3 h) and Cholesterol (1 mg/ml, 12 h) and LPS (1 µg/ml, 3 h) and MSU (200 µg/ml, 12 h). (n=3, Mean ±SD, **p ≤ 0.005, ***p ≤ 0.0005, Student's unpaired t test)

Supplementary Figure 7

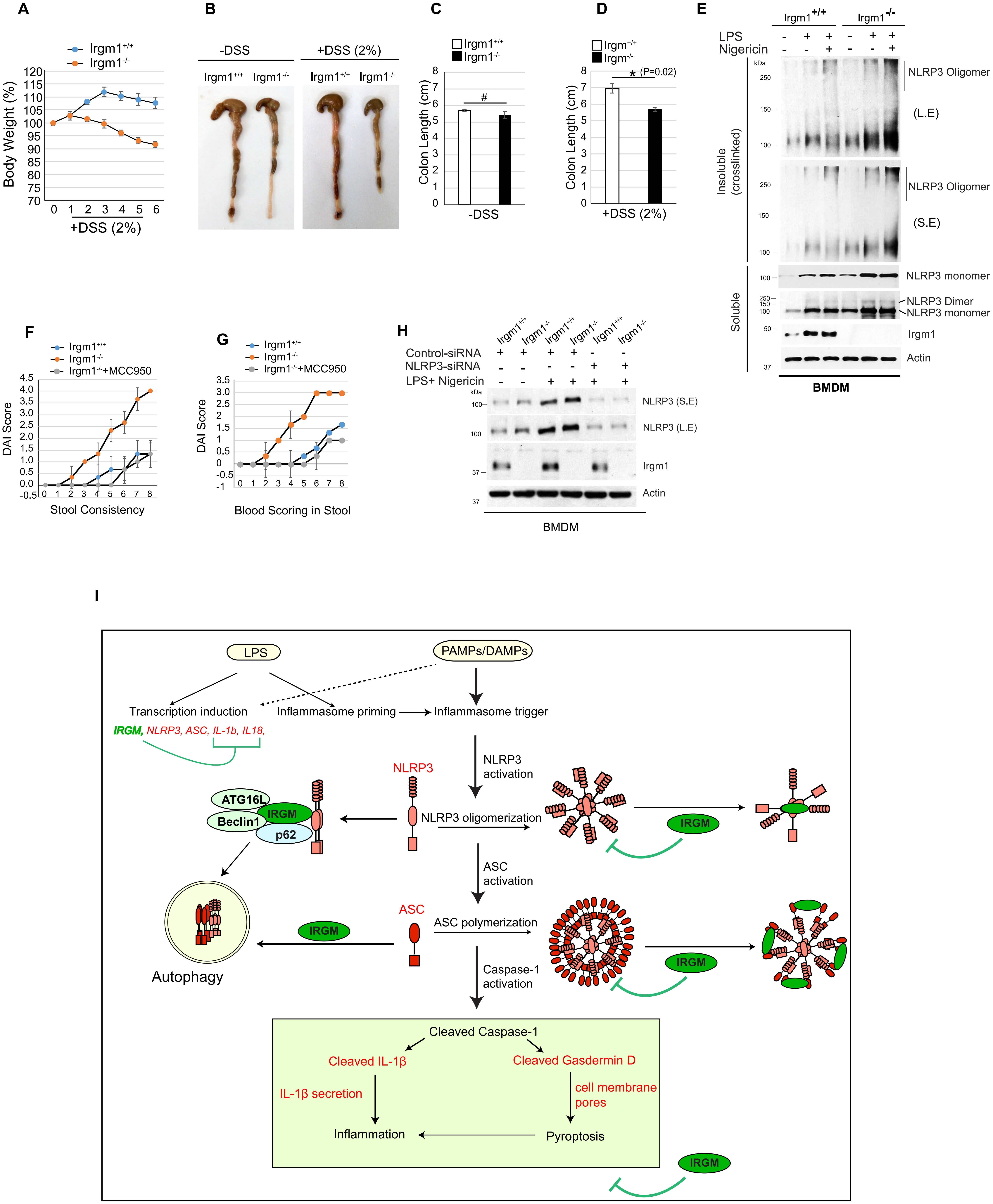

Figure S7. Irgm1 suppresses NLRP3 inflammasome in DSS-induced colitis mouse model, Related to Figure 7. (A) A group of three male mice each with similar weight were administrated with DSS (2%) for five days and body weight was measured every day. Graph depicts average body weight change during the course of treatment and before sacrifice. (B) Representative pictures of colon of DSS treated and untreated wild type and Irgm1 knock out mice. (C, D) Graph depicts colon length of DSS treated and untreated wild type and Irgm1 knock out mice (n=3, Mean ±SD, #insignificant, *p ≤ 0.005, student t-test unpaired). (E) Western blotting analysis of cross-linked insoluble/soluble cell fraction from LPS and nigericin treated $Irgm1^{+/+}$ and $Irgm1^{-/-}$ BMDM's. (F, G) Diseases activity index (DAI) score as measured from (F) tool consistency and (G) rectal bleeding information of Irgm1 wild type and knockout mice treated with 2% DSS and 20 mg/Kg of MCC950. (H) Western blot analysis of NLRP3 knockdown efficiency checked in BMDM treated with LPS (1 µg/ml, 3 h) and nigericin (5 µM, 30 min) (related to Figure 7O and 7P) from Irgm1 wild type and Knockout mice. In all panels, S.E, Short exposure; L.E, Long exposure. (I) This figure depicts the model of IRGM-mediated negative regulation of NLRP3 inflammasome activation. The data suggests that IRGM expression is induced by certain PAMPs and DAMPS and this increased expression of IRGM is important to keep under control the surge of excess inflammation provoked by the same PAMPs/DAMPs. One mechanism by which IRGM controls inflammation is by suppressing NLRP3 inflammasome. IRGM interacts with inflammasome components NLRP3 and ASC and reduces their oligomerization. Further, the IRGM mediates the autophagic degradation of NLRP3 and ASC. NLRP3 is degraded by IRGM in p62-dependent manner. By reducing the total amount and also by impeding the oligomerization capacity of inflammasome components, IRGM suppresses the inflammasome activation leading to lesser cleavage of Caspase-1, IL-1β and Gasdermin-D. Thus, inhibiting the process of pyroptosis and protecting the cells from surge of inflammation. This work is validated in mice model of colitis.
